# Implanted, Wireless, Self‐Powered Photodynamic Therapeutic Tablet Synergizes with Ferroptosis Inducer for Effective Cancer Treatment

**DOI:** 10.1002/advs.202302731

**Published:** 2023-11-13

**Authors:** Pingjin Zou, Rui Lin, Zengyi Fang, Junyang Chen, Hongye Guan, Jie Yin, Zhiheng Chang, Lili Xing, Jinyi Lang, Xinyu Xue, Meihua Chen

**Affiliations:** ^1^ School of Medicine University of Electronic Science and Technology of China Chengdu 610054 China; ^2^ Department of Radiation Oncology Radiation Oncology Key Laboratory of Sichuan Province Sichuan Clinical Research Center for Cancer Sichuan Cancer Center Sichuan Cancer Hospital & Institute Affiliated Cancer Hospital of University of Electronic Science and Technology of China Chengdu 610042 China; ^3^ School of Physics University of Electronic Science and Technology of China Chengdu 611731 China; ^4^ Chengdu University of Traditional Chinese Medicine Chengdu 611137 China; ^5^ School of Computer Science and Engineering University of Electronic Science and Technology of China Chengdu 611731 China

**Keywords:** ferroptosis inducers, implanted tablet, photodynamic therapeutic tablet, self‐powered unit, synergistic therapy, wireless power unit

## Abstract

The effective and targeted treatment of resistant cancer cells presents a significant challenge. Targeting cell ferroptosis has shown remarkable efficacy against apoptosis‐resistant tumors due to their elevated iron metabolism and oxidative stress levels. However, various obstacles have limited its effectiveness. To overcome these challenges and enhance ferroptosis in cancer cells, we have developed a self‐powered photodynamic therapeutic tablet that integrates a ferroptosis inducer (FIN), imidazole ketone erastin (IKE). FINs augment the sensitivity of photodynamic therapy (PDT) by increasing oxidative stress and lipid peroxidation. Furthermore, they utilize the Fenton reaction to supplement oxygen, generating a greater amount of reactive oxygen species (ROS) during PDT. Additionally, PDT facilitates the release of iron ions from the labile iron pool (LIP), accelerating lipid peroxidation and inducing ferroptosis. In vitro and in vivo experiments have demonstrated a more than 85% tumor inhibition rate. This synergistic treatment approach not only addresses the limitations of inadequate penetration and tumor hypoxia associated with PDT but also reduces the required medication dosage. Its high efficiency and specificity towards targeted cells minimize adverse effects, presenting a novel approach to combat clinical resistance in cancer treatment.

## Graphical abstract

An implanted, wireless, and self‐powered photodynamic therapeutic tablet that integrates a ferroptosis inducer imidazole ketone erastin (IKE) enhances ferroptosis and achieves a tumor inhibition rate of over 85%. This tablet promotes the generation of reactive oxygen species (ROS) during photodynamic therapy (PDT) by harnessing the Fenton reaction. It reduces IKE dosage and minimizes adverse effects, representing an innovative and effective treatment option for refractory tumors.

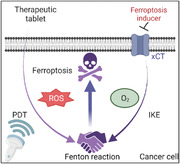

## 1 Introduction

Non‐small cell lung cancer (NSCLC) is the leading cause of cancer‐related mortality despite recent advancements in diagnosis and treatment,^[^ ^1^, ^2^ ^]^ innate and acquired drug resistance in cancer patients remains a significant challenge.^[^ ^3^, ^4^, ^5^ ^]^ To overcome drug resistance, researchers are currently exploring innovative techniques that might trigger cancer‐specific cell death.^[^ ^6^, ^7^, ^8^ ^]^ Ferroptosis,^[^ ^9^, ^10^ ^]^ a newly identified form of programmed cell death (PCD), is initiated by the accumulation of lipid peroxides in response to excessive iron ions, and its effectiveness in eliminating cells resistant to apoptosis, such as those with a high mesenchymal status or chemotherapy resistance, is due to unique features such as metabolic reprogramming, genetic mutations, and heightened oxidative stress in tumor cells.^[^ ^11^, ^12^, ^13^, ^14^ ^]^ Therefore, taking NSCLC as an example, selectively inducing ferroptosis holds promise as an effective cancer treatment approach.^[^ ^15^, ^16^ ^]^

Erastin is a classic ferroptosis inducer (FIN) due to its ability to induce ferroptosis by inhibiting the cystine‐glutamate antiporter (system Xc^−^ or xCT) and biosynthesis of glutathione (GSH), which leads to the accumulation of intracellular reactive oxygen species (ROS).^[^ ^14^, ^17^ ^]^ Nonetheless, its application is hindered by limitations in terms of efficacy, selectivity, and metabolic stability.^[^ ^18^ ^]^ To overcome these challenges, researchers have developed an erastin derivative known as imidazole ketone erastin (IKE). Animal studies have demonstrated that IKE exhibits improved metabolic stability, water solubility, and anticancer properties.^[^ ^18^, ^19^, ^20^ ^]^ However, the antitumor efficacy of erastin analogs used in isolation remains limited, necessitating further optimization to enhance their effectiveness.^[^ ^21^, ^22^, ^23^ ^]^

Photodynamic therapy (PDT) is a clinically approved, non‐invasive treatment that utilizes a photosensitizer to absorb specific wavelengths of light at the tumor site, generating ROS and cell death through apoptosis or ferroptosis.^[^ ^24^, ^25^ ^]^ This makes it an attractive option for targeted and combination therapy due to cancer cells' higher metabolic rate, and unique tumor microenvironment (TME), which enable selective uptake of the photosensitizer by cancer cells.^[^ ^26^ ^]^ Furthermore, PDT has minimal toxicity and lacks intrinsic or acquired resistance mechanisms associated with other cancer treatments. However, the clinical application of PDT is affected by the depth of light penetration and the hypoxic TME.^[^ ^27^, ^28^, ^29^, ^30^ ^]^ In our previous work, we developed a self‐powered implantable PDT tablet for treating deep tumors, which overcomes the limitations of conventional PDT and extends its clinical applications using the piezoelectric effect. ^[^ ^31^ ^]^

**Figure 1:**
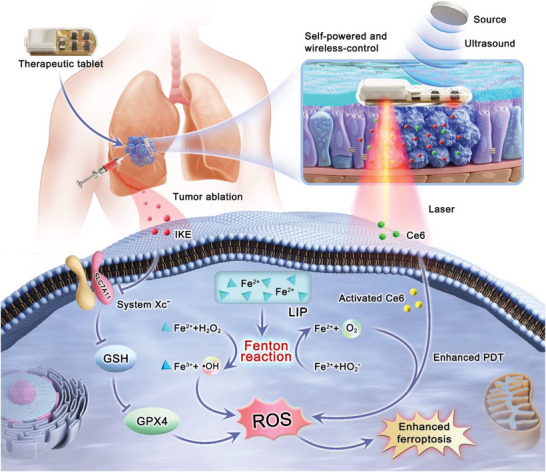
Ferroptosis inducer IKE combined with photodynamic therapy and underlying mechanisms for cancer treatment. Ferroptosis is induced in tumor cells by IKE via local inhibition of the SLC7A11‐GSH‐GPX4 signaling axis. In parallel, iron ions accumulate in the body, and via the Fenton reaction, improve the cellular hypoxic environment. The therapeutic tablet is minimally invasively implanted into the primary lung cancer in situ, and the µLEDs are driven to work by the action of ultrasound to activate the photosensitizer chlorin e6 (Ce6) using the oxygen supplemented by IKE for enhance photodynamic therapy, generate ROS, consume GSH. Lung cancer cells can be killed with ferroptosis because of the accumulation of ROS, which form lipid peroxides.

In this study, we optimized the design of the tablet to enhance its efficiency, sensitivity, and stability in generating ROS and depleting GSH. The PDT tablet was combined with the ferroptosis inducer, IKE, which inhibited cystine uptake mediated by solute carrier family 7 member 11 (SLC7A11), leading to further GSH depletion, glutathione peroxidase 4 (GPX4) inactivation, and lipid ROS accumulation. The combination of PDT tablet and IKE synergistically induced ferroptosis in cancer cells, providing a novel and efficient strategy for cancer treatment. FINs can increase the sensitivity of PDT by promoting oxidative stress and lipid peroxidation, and by utilizing the Fenton reaction to supplement oxygen to produce more ROS.^[^ ^32^ ^]^ PDT can also promote the release of iron ions from the labile iron pool (LIP), accelerating lipid peroxidation and inducing ferroptosis (**Figure** **1**). This combined treatment approach addresses the challenges of inadequate oxygen supply and limited treatment depth during PDT, and reduces the medication dosage required for FINs. Furthermore, this approach offers high efficacy and tumor cell specificity, protects normal cells, and overcome drug resistance, resulting in improved treatment effectiveness, patient compliance, and prognosis.

## 2 Results and Discussion

### 2.1 Bioengineering and Characterization of PDT Therapeutic Tablet

**Figure 2:**
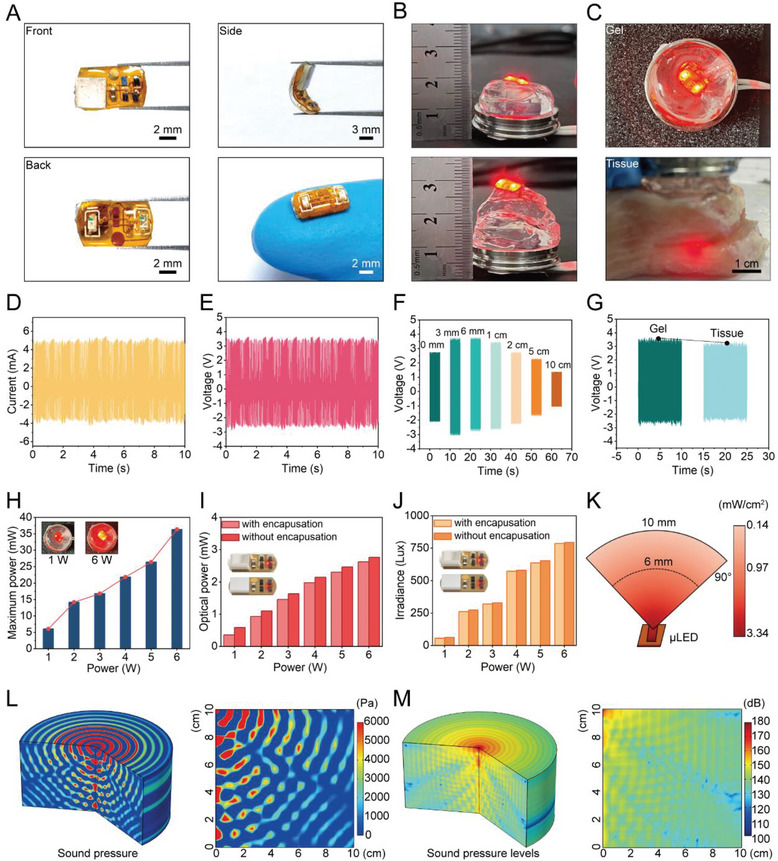
Characterization of photodynamic therapy tablet. (A) Appearance and flexibility of therapeutic tablet. (B) The deep operation of the tablet. (C) The tablet's operational status in different medium. (D) The current of the self‐powered unit in the tablet. (E) The voltage of the self‐powered unit in the tablet. (F) The voltage of the unit in different depth. (G) The output of the tablet in gel and tissue. (H) The output power of the unit under different ultrasound power. (I) The optical power of the tablet (with and without encapsulation) under different ultrasound power. (J) The therapeutic tablet's irradiance (with and without encapsulation) under varying ultrasonic power. (K) Simulation of the optical power of tablet with different depths. (L) Simulation of the sound pressure of tablet of varying depths. (M) Simulation of the sound pressure levels of tablet with different depths.

The tablet structure comprises a wireless power unit, µLED illuminant, and control circuit (**Figure** **2**A), and the preparation process is shown (Figure S1, Supporting Information). The wireless power unit receives ultrasound energy and converts it into electricity. Two 660 nm µLEDs deliver light for PDT. The control circuit on the top layer of the flexible Kapton film includes an independent rectifying circuits in a two‐sided etching configuration. Lead zirconate titanate (PZT) piezoelectric ceramics is connected to the top layer of the flexible circuit board, while two 660 nm µLEDs are connected to the bottom layer. The tablet is encapsulated in a biocompatible flexible polymer (polydimethylsiloxane, PDMS). It has a side length of approximately 5 mm × 8 mm, a thickness of 3 mm, and weighs 0.44 g. The piezoelectric generator is square piece, about 4 mm × 4 mm × 1 mm, with a resonant frequency of 500 kHz. The µLEDs have an area of approximately 1 mm × 0.5 mm. The miniaturized tablet enables minimally invasive surgery and a wireless power supply. Piezoelectric generator collects sound energy, convert it into electric energy, supply power, and control the intensity of µLED units.^[^ ^33^, ^34^ ^]^ To ensure good biocompatibility and avoid the generation of piezoelectric catalysis during the treatment process,^[^ ^35^, ^36^ ^]^ the PZT is encapsulated using PDMS,^[^ ^37^, ^38^ ^]^ which also prevents direct contact with the surrounding tissues.

We tested the tablet's output performance using a medical ultrasound gel to simulate tissue and an ultrasound source placed below the gel (Figure 2B; and Figure S2, Supporting Information). The output piezoelectric voltage and current under specific ultrasound waves and ambient conditions were approximately 3.47 V and 4.83 mA, respectively (Figure 2D,E). The tablet can typically work even at a depth of 50 mm under the skin, as the output voltage of the piezoelectric unit remained stable at different distances (Figure 2F). We also evaluated the output performance of the piezoelectric unit in various media (Figure 2C,G). The output piezoelectric voltage was 3.62 V and 3.44 V in medical ultrasound gel and a combination of medical ultrasound gel and pork media, respectively. Our results demonstrate that the tablet can provide wireless power and effective PDT for deep‐seated tumors.

When ultrasound penetrates the interface between the gel and the tissue, the attenuation is small, and the ultrasound is easily transferred to the tissue, and the output of the piezoelectric unit is basically unchanged. We found that changing the ultrasonic source's power can change the piezoelectric unit's output power (Figure 2H). The light irradiation performance of the tablet was also evaluated (Figure 2I). The electric energy output by the piezoelectric unit was used to activate the µLED, realizing the wireless control of lighting by using ultrasound and controlling the treatment dose of the system by adjusting the light intensity and time. The optical power of encapsulated and unencapsulated tablets under different ultrasound source powers was measured, and the results showed that the PDMS encapsulation had high visible light transparency and hardly affected the light irradiation (Figure 2J). We also measured the operation temperature (Figure S3A, Supporting Information), output of the unit related to the load resistance of the circuit (Figure S3B, Supporting Information), and corresponding output voltage and current (Figure S3C, Supporting Information). In terms of biological safety, we conducted further tests on the impact of the therapeutic tablet on local tissue (Figure S4, Supporting Information). After operating continuously for 1 hour, the skin tissue exposed to the ultrasound probe increased only from 23.2°C to 26°C, while the muscle tissue containing the tablet showed a minimal increase of less than 2°C (23.4°C to 25.2°C), demonstrating that the system does not cause any thermal damage to the local tissue.

Our study also measured the light transmission ex vivo using pork tissue. The light intensity emitted by the µLED was measured at different tissue thicknesses, and the results showed that red light attenuates by 70% when passing through 6 mm of tissue (Figure 2K). The light intensity attenuated from 3.34 to 0.97 mW/cm^2^. When treating large tumor tissue, the treatment time can be extended as needed to obtain a sufficient dose of therapeutic light intensity. We also measured the penetration depth of ultrasonic waves (Figure 2L,M). To simulate the distribution of ultrasonic sound pressure, we used water as a medium because it has water similar acoustic properties to human soft tissue (Z water = 1.48 MRayl, Z tissue = 1.63 MRayl, mean value for human tissue), we use water as a medium to simulate the distribution of ultrasonic sound pressure. The results show that ultrasound can be transmitted to a location below 10 cm of the medium for deep treatment.

### 2.2 Combining Ferroptosis Inducer IKE and PDT Therapeutic Tablet Enhance Efficacy In Vitro

**Figure 3:**
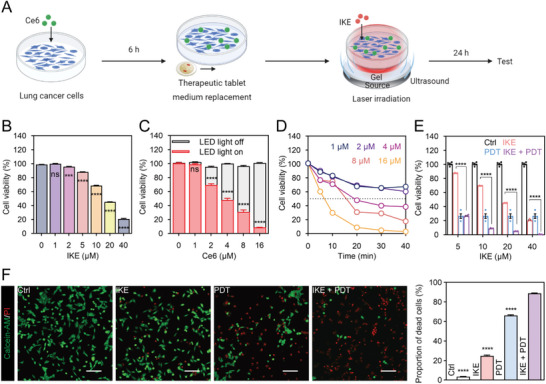
IKE combined with PDT therapeutic tablet could improve antitumor effect in vitro. (A) Illustrative description of treatments and experiments in vitro. (B) Cell viability of different concentration of IKE treatment by CCK‐8 assay. (C) The influence of the photosensitizer Ce6 on cell viability in the presence and absence of light. (D) Optimal parameter screening of therapeutic tablets for PDT. (E) Effect of different concentrations of IKE combined with PDT (8 µM Ce6, 20 min) on the activity of LLC cells. (F) IKE combined with PDT could cause more cell death by Calcein‐AM/PI fluorescence staining and corresponding statistical analysis. Scale bars, 50 µm. Data are presented as means ± SEM, *n* = 4. Ordinary one‐way ANOVA with multiple comparisons, not significant (ns), *P* ≥ 0.05; ^***^P ≤ 0.001; ^****^P ≤ 0.0001. (A) Created with BioRender.com, agreement number (DF25NGHDVW).

The in vitro efficacy of the ferroptosis inducer, IKE, with PDT via a therapeutic tablet was evaluated in three distinct cancer cell lines: human non‐small cell lung cancer cells A549 and H1299, and mouse Lewis lung cancer cell LLC. Schematic illustration of the experimental procedure is shown in Figure 3A.

The cell‐counting‐kit‐8 (CCK‐8) assay was utilized to determine the inhibition concentrations of lung cancer cell activity by IKE. All three cell lines survived when treated with 1 µM, 2 µM, and 5 µM IKE. Cell activity was inhibited only above 10 µM, and at 20 µM concentration, the viability of LLC, A549, and H1299 cells reduced to 45%, 52%, and 52%, respectively (Figure 3B; and Figure S5, Supporting Information). In order to investigate the therapeutic tablet with PDT, we tested varying concentrations of Ce6 photosensitizer, which had minimal to no cytotoxic effects without ultrasound activation. Before this, we carried out tests on the absorption spectrum and distribution state of Ce6 in the cell culture medium (Figure S6, Supporting Information), assessed the extent of cellular uptake (Figure S7, Supporting Information), and also demonstrated the non‐toxicity of Ce6 itself (Figure S8, Supporting Information). Upon illumination, the viability of lung cancer cells decreased in a dose‐dependent manner as the concentration of Ce6 increased (Figure 3C; and Figure S9, Supporting Information). The optimal photodynamic conditions of the therapeutic tablet were also examined, and PDT was observed to be more effective with an increase in the concentration of photosensitizer and treatment duration (Figure 3D; and Figure S10, Supporting Information). After 20 minutes, a decrease in cell viability was observed with various Ce6 concentrations, where all three cell lines showed 30% activity (LLC: 30.75%, A549: 27.59%, and H1299: 31.1%) at 8 µM Ce6. The impact of PDT operating conditions on the efficacy of IKE treatment was then examined (Figure 3E; and Figure S11, Supporting Information). At the 10 µM IKE, IKE plus PDT treatment group, LLC and H1299 cellular viability was reduced by 90%, and A549 by nearly 85%. The therapeutic efficacy improved by 7.8, 8.2, and 4.6‐fold, respectively. We observed that PDT significantly improved the efficacy of IKE and decreased the required dose. The cytotoxicity of different treatments was also verified via the fluorescence imaging of calcein acetoxymethyl ester (Calcein‐AM) and propidium iodide (PI). The combination treatment group exhibited higher proportion of cell death than the individual IKE and PDT groups (Figure 3F; and Figure S12, Supporting Information).

These findings suggest that the combination of ferroptosis inducer and PDT therapeutic tablet showed promising results in inducing cell death in vitro, and can potentially provide a novel therapeutic strategy for cancer treatment.

### 2.3 Mechanisms Induced by IKE in Conjunction with PDT Therapeutic Tablet

**Figure 4:**
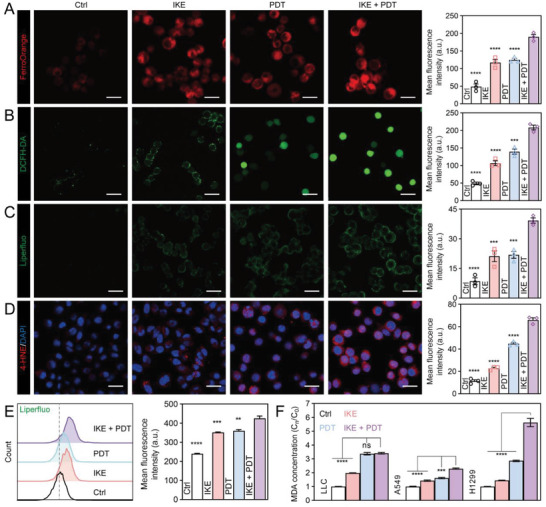
Ferroptosis induction of lung cancer cells by IKE combined with PDT therapeutic tablet. (A) Labile iron ion levels were detected by FerroOrange fluorescence probe and corresponding quantitative analysis. Scale bars, 25 µm. (B) Total ROS generation in lung cancer cells after different treatments were detected by confocal microscopy and corresponding quantitative analysis. Scale bars, 50 µm. (C) Liperfluo fluorescent staining and statistical analysis on cells treated with different regimens. Scale bars, 50 µm. (D) 4‐HNE (red) fluorescent staining and statistical analysis. Scale bars, 50 µm. (E) Flow cytometric detection and statistical analysis of liperfluo as a measure of lipid peroxidation in lung cancer cells. (F) MDA kit‐based relative quantification of intracellular malondialdehyde levels. Data are presented as means ± SEM, *n* = 3. Ordinary one‐way ANOVA with multiple comparisons, not significant (ns), *P* ≥ 0.05; ^**^P ≤ 0.01; ^***^P ≤ 0.001; ^****^P ≤ 0.0001.

To confirm whether the combination therapy of IKE and PDT induces ferroptosis, we conducted various assays on cells treated with different regimens to assess lipid peroxidation, labile iron regulation, ROS biology, and lipid peroxidation. The FerroOrange fluorescent probe^[^ ^39^ ^]^ was used to detect ferrous ions (Fe^2+^) in each treatment group, and confocal imaging showed that the IKE + PDT group had the strongest signal (Figure 4A; and Figure S13, Supporting Information), indicating an increase in free ferrous ions that may promote the Fenton reaction and oxidative stress. We also used the 2′,7′‐dichlorodihydrofluorescin diacetate (DCFH‐DA)^[^ ^40^ ^]^ probe to measure ROS levels and found that the IKE + PDT group had higher levels of ROS compared to the other treatment groups (Figure 4B; and Figure S14, Supporting Information). The Liperfluo probe^[^ ^40^ ^]^ was used to assess cellular lipid peroxidation, and both fluorescence imaging (Figure 4C) and flow cytometry (Figure 4E; and Figure S15, Supporting Information) showed strong signals in all three treatment groups, with the strongest signal in the IKE + PDT group. 4‐hydroxynonenal (4‐HNE) and malondialdehyde (MDA), the lipid peroxidation end products, were also higher in the IKE + PDT group (Figure 4D,F). These results suggest that the combination therapy of IKE and PDT induces ferroptosis by increasing labile iron levels, promoting ROS production, and inducing lipid peroxidation.

**Figure 5:**
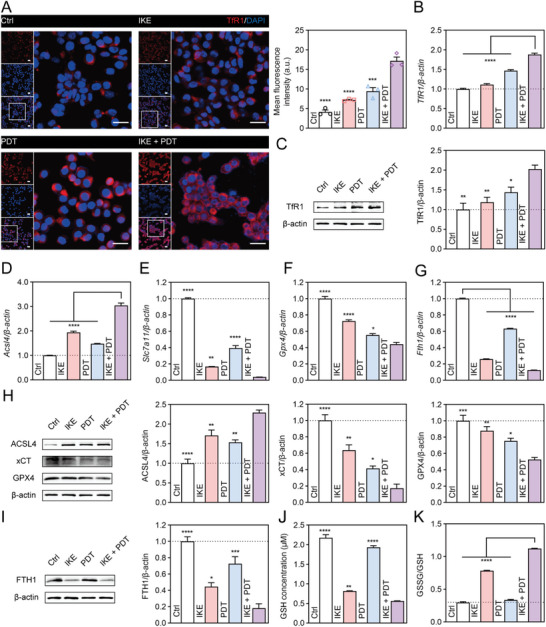
Ferroptosis induction of lung cancer cells by IKE combined with PDT therapeutic tablet. (A) TfR1 (red) fluorescent staining and statistical analysis. Scale bar, 25 µm (long) and 150 µm (short). (B) Relative expression levels of *TfR1* by real‐time fluorescence quantitative PCR. (C) Western blot assay of TfR1 expression the cell level in different treatment groups and statistical analysis. (D) Relative expression levels of *Acsl4* by real‐time fluorescence quantitative PCR. (E) Relative expression levels of *Slc7a11* by real‐time fluorescence quantitative PCR. (F) Relative expression levels of *Gpx4* by real‐time fluorescence quantitative PCR. (G) Relative expression levels of *Fth1* by real‐time fluorescence quantitative PCR. (H) Western blot assay of ACSL4, xCT, and GPX4 expression in different treatment groups and statistical analysis. (I) Western blot assay of FTH1 expression in different treatment groups and statistical analysis. (J) The concentration of GSH in different treatment groups. (K) The ratio of GSSG/GSH in different treatment groups. Data are presented as means ± SEM, *n* = 3. Ordinary one‐way ANOVA with multiple comparisons, ^*^ *P <* 0.05; ^**^ *P ≤* 0.01; ^***^ *P ≤* 0.001; ^****^ *P ≤* 0.0001.

Furthermore, in the IKE + PDT group, transferrin receptor protein 1 (TfR1) was found to upregulated, and its translocation to the cell surface can enhance cellular iron uptake, which is essential for the generation of ROS and lipid peroxidation (Figure 5A,B,C). We also examined the expression of ferroptosis‐related genes, including *Acsl4* (Figure 5D), *Slc7a11* (Figure 5E), *Gpx4* (Figure 5F), and *Fth1* (Figure 5G). We found that both IKE and PDT could dynamically regulate their expression. Furthermore, additional protein results confirmed these findings (Figure 5H,I). The higher expression levels of *SLC7A11* and *GPX4* in lung cancer tissues compared to normal tissues suggest that targeting the SLC7A11‐GSH‐GPX4 axis with the combination therapy of IKE and PDT may improve the prognosis of cancer patients (Figure S16, Supporting Information). We measured GSH levels in cells treated with different regimens and found that both the IKE and PDT groups led to GSH depletion, but the IKE + PDT group had a further reduction in GSH concentration (Figure 5J), increased the ratio of oxidized glutathione (GSSG) to GSH (Figure 5K), and disrupted the antioxidant balance.

Additionally, the combination therapy of IKE and PDT can downregulate hypoxia‐inducible factor 1, alpha subunit (*Hif1a*), improve the hypoxic microenvironment, and enhance the sensitivity of PDT (Figure S17, Supporting Information). Overall, our research results indicate that the combination therapy of IKE and PDT induces ferroptosis in cancer cells through multiple mechanisms, including disrupting the SLC7A11‐GSH‐GPX4 antioxidant signaling axis, depleting GSH levels, increasing labile iron ion content, promoting ROS production, and inducing lipid peroxidation.

### 2.4 Therapeutic Effects of Combination Treatments in LLC Lung Cancer Model

**Figure 6:**
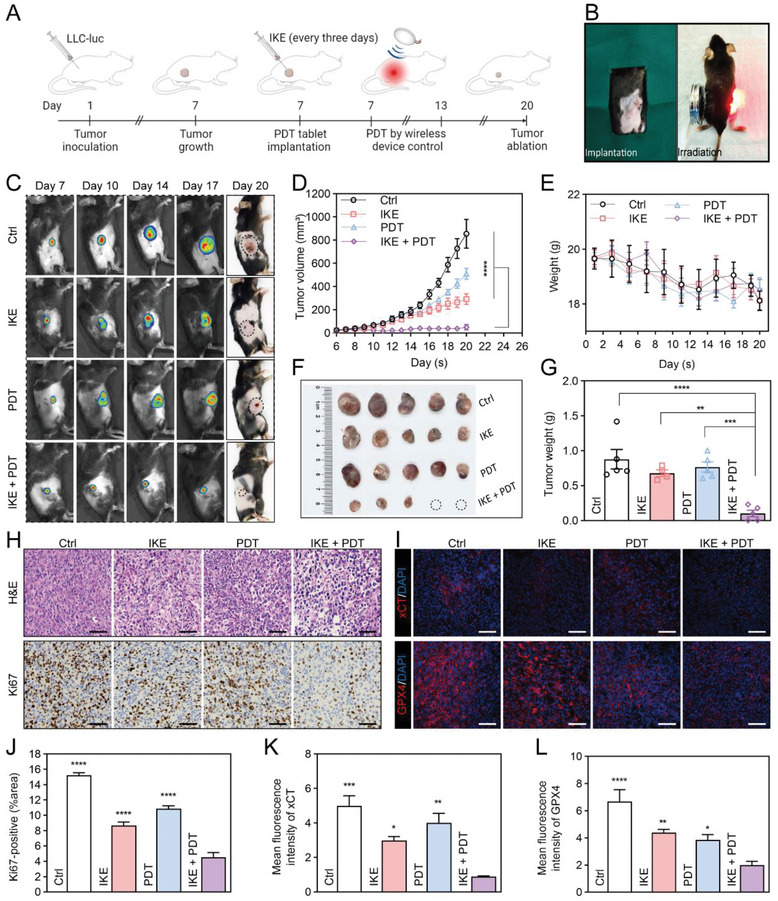
Therapeutic efficacy of IKE combined with PDT therapeutic tablet against LLC subcutaneous tumor model. (A) Schematic illustration of the experimental procedure. (B) The implantation and working of therapeutic tablet. (C) In vivo reprehensive fluorescence imaging of subcutaneous LLC‐luc tumor‐bearing C57BL/6 mice with different treatments. (D) Average tumor growth curves of all the groups. (E) Body weight changes after intertumoral injection of IKE, Ce6 with and without µLEDs irradiation over a period of 20 days. (F) Photographs of the tumors collected from each group (day 20). (G) Weight of the tumors collected from each group (day 20). (H) Representative H&E and Ki‐67 staining of tumor sections from the tumor tissues in different treatment groups (day 20). Scale bars, 50 µm. (I) Representative xCT and GPX4 immunofluorescence staining of tumor sections from the tumor tissues in different treatment groups (day 20). Scale bars, 50 µm. (J) The corresponding immunohistochemical index quantitative analysis of Ki‐67. (K) The corresponding quantitative analysis of the mean fluorescence intensity (a.u.) of xCT. (L) The corresponding quantitative analysis of the mean fluorescence intensity (a.u.) of GPX4. Data are presented as means ± SEM, *n* = 5. Ordinary one‐way ANOVA with multiple comparisons, ^*^P < 0.05; ^**^P ≤ 0.01; ^***^P ≤ 0.001; ^****^P ≤ 0.0001. (A) Created with BioRender.com, agreement number (IV25MOQ6G0).

We investigated the therapeutic efficacy of the PDT therapeutic tablet combined with IKE in LLC‐luc lung cancer‐bearing mice (Figure 6A). For this study, we implanted a PDT therapeutic tablet deeply inside the tumor when it reached 35–50 mm^3^ after 7 days (refer to Figure S18, Supporting Information for the specific procedural details). Following intra‐tumoral injection of 10 µg Ce6 for 6 hours, a wireless ultrasound probe was used to provide deep tumor treatment for 40 minutes per session over seven days. During this period, 50 mg^−1^kg of IKE was injected into the tumor every three days, for a total of three injections. This dosage is smaller than the standard dose.^[^ ^18^, ^41^ ^]^ The implanted device and ultrasound‐based treatment procedures are displayed (Figure 6B; and Movie S1, Supporting Information). We evaluate the efficacy of therapy pre, during, and post‐treatment using an animal in vivo imaging system to conduct fluorescence imaging of the mice on days 7, 10, 14, and 17 after tumor inoculation. On day 20, we analyzed photographs of mice with tumors that received various treatments (Figure 6C).

**Figure 7:**
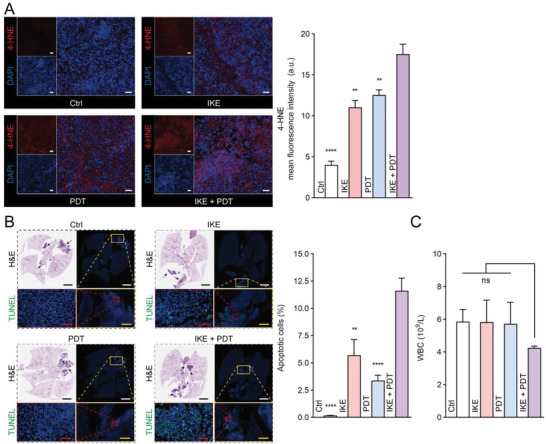
Therapeutic efficacy of IKE combined with PDT therapeutic tablet against LLC subcutaneous and in‐situ tumor model. (A) 4‐HNE fluorescent staining results in tumor tissue and quantitative analysis. *n* = 3. Scale bar, 100 µm. (B) Panoramic scan of LLC in situ lung cancer and TUNEL fluorescence staining. *n* = 5. Scale bar, 3 mm (black and white), 500 µm (yellow), 50 µm (red). (C) Analysis of peripheral blood leukocyte count, *n* = 3. Data are presented as means ± SEM. Ordinary one‐way ANOVA with multiple comparisons, not significant (ns), *P* ≥ 0.05; ^**^ *P* ≤ 0.01; ^****^ *P* ≤ 0.0001.

Compared to the control group, neither IKE nor PDT alone significantly suppressed tumor growth, while the combination of IKE and PDT resulted in a remarkable reduction of the average tumor volume by 88.0% (Figure 6D; and Figure S19, Supporting Information). On day 20, the excised tumors were weighed and photographed (Figure 6F). The average tumor weight also fell by 88.4% in the IKE + PDT group compared to the control group (Figure 6G). Hematoxylin and eosin (H&E) staining experiments confirmed the decreased tumor cell density following IKE + PDT therapy (Figure 6H), and Ki‐67 immunohistochemical staining revealed the least amount of proliferation in this group (Figure 6J). The immunofluorescence images of xCT and GPX4 in tumor tissue (Figure 6I), along with their corresponding statistical results (Figure 6K,L), also confirmed the regulation of the SLC7A11‐GPX4 signaling axis by IKE + PDT. Furthermore, we observed an increase in 4‐HNE levels within the tumor tissue (Figure 7A). No significant changes of body weight (Figure 6E), routine blood tests (Figure S20, Supporting Information), biochemical parameters (Figure S21, Supporting Information), and histomorphology of major organs (Figure S22, Supporting Information) indicated that the treatment had fewer side effects and higher biosafety in vivo.

To further demonstrate the biological safety of the combined treatment approach, we co‐cultivated the therapeutic device with normal cells, human bronchial epithelial cells (HBE), and mouse embryonic fibroblasts (NIH3T3). Results from Calcein‐AM/PI staining indicated that the tablet had low toxicity on normal cells (Figure S23A, Supporting Information). Meanwhile, we implanted the therapeutic medication into the thoracic cavity of mice and rats through minimally invasive surgery and gave different interventions to observe their biocompatibility and adverse reactions. The implanted tablet and ultrasound‐based treatment procedures are displayed (Movie S2, Supporting Information). H&E staining of lung tissue showed that the therapeutic medication did not significantly affect normal lung tissue (Figure S23B,C, Supporting Information). Diff‐Quik staining of rat bronchoalveolar lavage fluid (BALF) showed that alveolar macrophages were still the main cell type present, and no significant neutrophils or immune cells were observed, indicating that adverse reactions caused by local injection of IKE and Ce6 were minimal (Figure S24, Supporting Information).

Next, we constructed an LLC‐luc lung cancer in situ model and gave different interventions to further evaluate the biological safety and anti‐tumor effects. The implanted tablet and ultrasound‐based treatment procedures are displayed (Movie S3, Supporting Information). TUNEL staining showed that IKE and PDT‐induced cell death was targeted and did not have significant effects on normal cells and tissue, while the IKE + PDT group had a stronger anti‐tumor effect (Figure 7B). Results of the white blood cell counts reflected that the combined treatment did not cause systemic inflammatory reactions (Figure 7C).

The results of our study demonstrate that the combination of IKE and PDT considerably enhances the antitumor efficacy in malignant tumors while maintaining a high level of biological safety.

Combining PDT with ferroptosis has emerged as an innovative approach to modulate intracellular redox homeostasis, enhance cancer cell susceptibility to oxidative stress, and elicit synergistic anticancer effects, holding significant promise for anticancer treatment.^[^ ^42^, ^43^ ^]^ Within this strategy, the coalescence of PDT with small molecule ferroptosis inducers has garnered attention. Zhu and colleagues developed an erastin‐Ce6 self‐assembling nanoplatform, demonstrating heightened levels of tumor cell ROS and improved anticancer efficacy compared to single Ce6‐based PDT. ^[^ ^44^ ^]^ Similarly, Du et al. utilized exosomes (Er/RB@Exos^CD47^) for the targeted delivery of erastin (Er) and Rose Bengal (RB) to tumor sites, exhibiting effective evasion of tumor clearance through CD47‐mediated mechanisms and promising in vivo anticancer activity. ^[^ ^45^ ^]^ By utilizing ferroptosis inducers and photosensitizers at reduced doses, the potential side effects can be minimized. However, despite the therapeutic efficacy demonstrated by these approaches, their clinical translation is hindered by intricate manufacturing processes and limited scalability for mass production.

In this study, we have developed a self‐powered PDT device using PZT and biocompatible PDMS, combined with FIN (IKE), offering enhanced selectivity and metabolic stability. This design enables facilitates the attainment of superior antitumor effects with ease and stability, thereby presenting promising prospects for clinical translation. Leveraging the localization and selectivity provided by the light source of the therapeutic tablet, the inherent selectivity of the photosensitizer, and the localized application of FIN, we have achieved heightened selectivity towards tumor tissue, minimizing damage to normal tissue. Nevertheless, our future focus lies in the precise targeting of tumor cells. For instance, the incorporating tumor‐specific ligands or antibodies, or the implementation of strategies to facilitate precise drug release, will further enhance the targeted effect on tumor cells while minimizing potential off‐target effects on healthy cells.

## 3 Conclusion

In summary, the wireless and self‐powered photodynamic therapeutic tablet can be implanted into deep‐seated tumors through minimally invasive surgery, enabling targeted treatment in conjunction with the ferroptosis inducer, IKE. This combined approach offers a novel strategy to effectively combat resistance in cancer treatment by inducing ferroptosis with high specificity. The synergistic treatment approach capitalizes on the altered metabolic characteristics and microenvironment of tumor cells, enabling precise targeting while minimizing harm to healthy tissues. Besides the current lung cancer research model, we are actively investigating the therapeutic effects of this approach in other types of cancer, such as breast cancer. Moreover, the integration of tumor‐targeting elements, such as a tumor‐specific ligands or antibodies, along with drug delivery systems (DDS) like microneedles into therapeutic tablets, holds potential for reducing invasiveness, enhancing patient convenience, improving compliance, and advancing personalized cancer treatment platforms.

## 4 Experimental Section

### Materials

The PZT was obtained from SCH Technology Co. Ltd. The control circuit with 660 nm µLEDs was obtained from Inper LLC. The PDMS Sylgard184 was purchased by Dow Corning. Lung cancer cell line LLC, A549, and H1299 were obtained from the Cell Bank of Chinese Academy of Sciences and cultured in Dulbecco's modified Eagle's medium (Yeasen, China) supplemented with 10% (v/v) fetal bovine serum (40 130, Yeasen, China). Chlorin e6 (Ce6) was obtained from Macklin Biochemical Technology Co., Ltd. (Shanghai, China). Imidazole ketone erastin (IKE) was purchased from Selleck Chemicals Co., Ltd. (Shanghai, China). Female BALB/c mice aged six to eight‐week were purchased from Beijing HFK Bioscience Co., Ltd. (www.hfkbio.com). Anti‐xCT (ab307601), GPX4 (ab125066), and 4‐HNE (ab46545) primary antibodies were purchased from Abcam (Shanghai, China). Anti‐FTH1 (A19544), TfR1 (A5865), ACSL4 (A6826), 4‐HNE (A24456), and β‐actin (AC026) primary antibodies were purchased from Abclonal (Wuhan, China). D‐Luciferin, sodium salt (40 902) was purchased from Yeasen (Shanghai, China).

### Therapeutic tablet preparation

The formulation of a therapeutic tablet was based on earlier work.^[^ ^31^ ^]^ The therapeutic tablet consisted mostly of a self‐powered unit, µLED illuminants, and a control circuit. The square‐shaped PZT (length = 4 mm, width = 4 mm, and thickness = 1 mm) was initially customized. The self‐powered unit was then pre‐cleaned with ethanol and deionized water before being dried naturally. Thirdly, the PZT and two 660 nm µLEDs were connected to the control circuit. The mixture of PDMS elastomer and cross‐linker with a mass ratio of 10:1 was then treated under vacuum for 5 minutes to eradicate air bubbles. The tablet was then encapsulated with a PDMS mixture and air‐dried.

### Characterization and measurements

Scanning electron microscopy was utilized to examine the structure and morphology of the materials (SEM, Zeiss Gemini 300). X‐ray diffraction was utilized to determine the crystal phase of piezoelectric material (XRD, D/max 2550 V, CuKa radiation). The self‐powered unit's piezoelectric output was evaluated using low noise voltage/current preamplifiers (Stanford Research Systems Model SR560/570). The µLED luminaire's light intensity was tested with an optical power meter (M100D, Thorlabs). The light irradiance was determined using a commercial meter (PM6612, Peak). Using a commercial meter, the temperature was measured (PM6530D, Peak).

### Cell Culture

Three cell lines, LLC, A549, and H1299 were cultured in DMEM or RPMI‐1640 medium (Yeasen, China) supplemented with 10% fetal bovine serum (Yeasen, China), 100 µg/ml streptomycin, and 100 U/ml penicillin (GibcoTM, Thermo Fisher Scientific, USA). Cells were then incubated at 37°C in a humidified chamber containing 5% CO_2_.

### Characteristics and preparation of Chlorin e6 (Ce6)

Ce6 is a second‐generation photosensitizer and penetrates deeper tissues and treats deeper tumors due to its higher selectivity for cancerous tissues, higher absorption coefficient, and longer wavelength of maximum absorption.^[^ ^26^, ^46^ ^]^ Ce6 can return to its ground state through fluorescence emission or convert to a triplet state through intersystem crossing after light absorption. The triplet state of Ce6 can produce ROS that damage cancer cells.^[^ ^47^ ^]^ 100 mg of Ce6 powder was completely dissolved in 16.7 ml of cell‐grade DMSO^[^ ^48^, ^49^ ^]^ under light‐avoiding conditions, and then filtered and sterilized. The solution was frozen at −20°C. Before use, it should be diluted to the appropriate concentration with PBS or cell culture medium.

### Uptake of Ce6 by cancer cells

1 × 10^5^ LLC cells were incubated in the dark with 2, 4, 8, 16, and 32 µM Ce6 prepared in serum‐free DMEM. After 2 hours of incubation, the cells were washed twice with PBS, and fresh culture medium was added. Then, confocal imaging was performed or the cells were digested for flow cytometry analysis.

### Cell Viability Assay

8 × 10^3^ cells were seeded per well in 96‐well plates, and grown for 24 hours for in vitro concentration measurements. After removing the culture media, 100 µl of culture media containing a gradient of Ce6 (1, 2, 4, 6, 8, and 16 µM) and erastin (1, 2, 5, 10, 20, and 40 µM) was added. Ctrl, IKE, PDT, and IKE + PDT hybrid therapy groups underwent viability assessments. After incubation for 6 hours, the PDT and IKE + PDT hybrid therapy groups were exposed to µLEDs (660 nm, 6 W) for 20 minutes. After 24 hours of incubation for cytotoxicity testing, cell viability was determined using the CCK‐8 assay (ABclonal Technology, China). Each cell population was plated thrice.

### Live/Dead Assay

The Calcein‐AM/PI Cell Viability/Cytotoxicity Assay Kit (Beyotime Biotechnology, China) was used for the live/dead assay. The assay used Calcein‐AM in combination with PI for dual fluorescence staining of both live and dead cells. Calcein‐AM‐stained live cells with green fluorescence, while PI‐stained dead cells with red fluorescence. To perform the assay, 250 µl of Calcein AM/PI assay working solution was added to each well of a 24‐well plate. The plate was then incubated for 30 min at 37°C, protected from light. After incubation, the staining effect was observed under a fluorescence microscope (Nikon).

### Measurement of Intracellular ROS levels

The Reactive Oxygen Species Assay Kit (Beyotime, China) was used to measure intracellular ROS levels. The kit used intracellular reactive oxygen species to oxidize non‐fluorescent 2′,7′‐dichlorofluorescein‐diacetate (DCFH‐DA) to generate fluorescent dichlorofluorescein (DCF). The level of intracellular ROS can be detected by measuring the fluorescence of DCF. Briefly, A549 (2 × 10^4^/well), H1299 (2 × 10^4^/well), and LLC (5 × 10^4^/well) cells were seeded in 24‐well plates and exposed to the four group treatments as described above. After treatment, the cells were incubated with DCFH‐DA for 20 min at 37°C and then observed using fluorescence microscopy (Nikon) and measured at 488 nm excitation and 525 nm emission by a fluorescence spectrophotometer.

### Detection of cellular GSH and GSSG

3 × 10^5^ LLC cells were seeded in a 6‐well plate and treated with different interventions. After 48 hours, the cell suspension was collected and subjected to 3 repeated freeze‐thaw cycles for subsequent experiments. The GSH and GSSG assay kits were used according to the manufacturer's instructions (Beyotime Biotechnology, China) to determine the concentration.

### Lipid Peroxidation Assay

To measure lipid peroxidation, cells were seeded in (confocal) dishes at a density of 1.5 × 10^5^ cells. After treatment, cells were incubated with Liperfluo (1 µmol/L) for 30 minutes, washed twice with 1 ml HBSS, and then 200 µl HBSS was added uniformly. Cells were collected for analysis using a BD FACSCanto II flow cytometer or imaged using a confocal laser scanning microscope. The excitation and emission wavelengths of the oxidized Liperfluo are 524 and 535 nm, respectively.

### Measurement of Malondialdehyde (MDA) level

To measure MDA levels, cells were cultured in 24‐well plates and treated to induce oxidative stress. We then collected cells according to the manufacturer's protocol, and used the supernatant for each measurement. Fluorescence intensity was detected with a BioTek Cytation 5 (Agilent) (Ex: 540 nm, Em: 590 nm). We calculated the MDA concentration in the samples from the standard curve of MDA.

### Quantitative real‐time PCR

Total RNA from cells was extracted by Trizol (Invitrogen) following the manufacture's introduction. The concentration of RNA was measured with a Nanodrop‐2000 spectrophotometer (Thermo Scientific, USA). For analysis of mRNA expression, 1 µg total RNA was reverse transcribed using EvoM‐MLV RT Mix Tracking Kit (AG11734, Accurate Biotechnology, China), and qPCR was performed using SYBR Green Premix Pro Taq HS qPCR Tracking Kit (AG11733, Accurate Biotechnology, China). *β‐actin* was used as a housekeeping gene and relative expression of the target gene was determined using the 2 ^‐ΔΔCt^ method.

### Western blotting (WB) analysis

Protein samples were extracted from the cells and lysed using RIPA buffer with protease inhibitor. The protein concentration was determined using a BCA assay kit. Equal amounts of protein were separated by SDS‐PAGE gel electrophoresis and transferred onto a PVDF membrane using wet transfer method. After transfer, the membrane was blocked with a blocking solution to prevent non‐specific binding. The membrane was then incubated with primary antibodies specific to the target proteins (1:1000) overnight at 4°C. After washing, the membrane was incubated with secondary antibodies conjugated to an enzyme dye for 1 hour at room temperature. Protein bands were visualized using chemiluminescence detection methods. The intensity of the protein bands was quantified using densitometry analysis software. The experiment was performed in triplicate, and representative images are shown.

### Ethical statement

Standard laboratory diets and water were provided to all mice. Standard laboratory conditions were used (21°C ± 2°C, 12 hours of darkness/12 hours of light) for housing the animals. All animal experiments including animal handling, care, and treatment were conducted in accordance with the Animal Study Guidelines of Sichuan Cancer Center (Approval Number: SCCHEC‐04‐2022‐010). We used the ARRIVE guidelines 2.0.

### Suppression of tumor growth in vivo

5 × 10^5^ LLC‐luc cells were subcutaneously inoculated into the right flank of mice to establish a homograft tumor model. When the tumors reached a size of approximately 35–50 mm^3^, mice were randomly assigned into four groups (n = 5/group): (i) control group; (ii) single IKE; (iii) single PDT; (iv) IKE + PDT hybrid therapy. Tablet implantation was performed for groups (iii) and (iv). Starting on day 7, 10 µg Ce6 was injected into the tumor for groups (iii) and (iv), and 6 hours later, light irradiation was administered for 40 minutes. The treatment period lasted seven days. Mice were weighed every two days, and the length and width of the tumors were measured using vernier calipers. Tumor growth was monitored using in vivo fluorescence imaging on days 7, 10, 14, and 17. At the end of the observation period (day 20), mice were euthanized, and blood was collected. Then tumors were excised, weighted, and photographed. For histology analysis, tumors and normal organs including the heart, liver, spleen, lung, and kidney were harvested for H&E staining, Ki‐67 immuno‐histochemistry staining, xCT, GPX4, and 4‐HNE immunofluorescent staining (for tumor tissues only).

### Biocompatibility evaluation of therapeutic tablet

In vitro, we co‐cultured the therapeutic tablets with normal HBE and NIH3T3 cells for 24 hours and performed Calcein‐AM/PI staining for live/dead cell detection. In vivo, we surgically implanted the therapeutic tablets into the thoracic cavity of mice and rats, and collected lung tissue for H&E staining.

### Biological safety of combination therapy

Therapeutic tablets were surgically implanted into the thoracic cavity of SD rats and randomly divided into four groups: the control group, IKE group, PDT group, and IKE + PDT group, for different interventions. On the 1st, 3rd, 7th, and 10th days after intervention, bronchoalveolar lavage fluid was collected for Diff‐Quik staining to observe the cell composition, and lung tissue was collected for HE staining.

### Suppression of tumor growth in situ

A suspension of 2 × 10^6^ LLC‐luc cells in 50 µl was mixed with low growth factor matrix gel at a ratio of 1:1 and implanted into the left lung to establish an in‐situ lung cancer model. After 3 days of tumor growth, live imaging was performed to confirm the successful establishment of the model. The mice were randomly divided into groups (*n* = 5), as in the subcutaneous tumor model. The IKE, PDT, and IKE + PDT groups were injected intrapleurally with IKE and/or Ce6, followed by 40 minutes of photodynamic therapy 6 hours later. Continuous treatment was administered for 3 days, and then lung tissues were collected for HE and TUNEL staining, and peripheral blood was collected for routine blood analysis.

### Statistical analysis

The Ordinary one‐way ANOVA with multiple comparisons was used to perform statistical analysis using GraphPad Prism 9 (GraphPad Software, Inc., California, USA). Statistically significant was concluded at ^*^ P < 0.05, ^**^ P ≤ 0.01, ^***^ P ≤ 0.001, ^****^ P ≤ 0.0001. Data are presented as the means ± SEM.

## Conflict of Interest

The authors declare no conflict of interest.

## Author Contributions

J.L., M.C., L.X. and X.X. conceived this idea. M.C., X.X., P.Z., R.L. and H.G. designed the experiments. P.Z., R.L., Z.F., J.C., J.Y. and Z.C. performed the experiments and data analysis. P.Z. and R.L. co‐wrote the paper. J.L., M.C., L.X. and X.X. reviewed and modified the paper.

## Supporting information

Supporting Information

Supplemental Movie 1

Supplemental Movie 2

Supplemental Movie 3

## Data Availability

The data that support the findings of this study are available from the corresponding author upon reasonable request.
