# Supplementary material for: Implanted, Wireless, Self‐Powered Photodynamic Therapeutic Tablet Synergizes with Ferroptosis Inducer for Effective Cancer Treatment
Source: Adv Sci (Weinh). 2023 Nov 13;10(36):2302731. doi: 10.1002/advs.202302731 (PMC10754143; doi:10.1002/advs.202302731)
Supplement: Supplementary file 1 — Supporting Information [file ADVS-10-2302731-s003.pdf]

ADVANCED SCIENCE
Open Access

Supporting Information

for *Adv. Sci.*, DOI 10.1002/advs.202302731

Implanted, Wireless, Self-Powered Photodynamic Therapeutic Tablet Synergizes with Ferroptosis Inducer for Effective Cancer Treatment

Pingjin Zou, Rui Lin, Zengyi Fang, Junyang Chen, Hongye Guan, Jie Yin, Zhiheng Chang, Lili Xing, Jinyi Lang, Xinyu Xue* and Meihua Chen**

Supporting Information

Implanted, Wireless, Self-Powered Photodynamic Therapeutic Tablet Synergizes with Ferroptosis Inducer for Effective Cancer Treatment

Pingjin Zou, Rui Lin, Zengyi Fang, Junyang Chen, Hongye Guan, Jie Yin, Zhiheng Chang, Lili Xing, Jinyi Lang*, Xinyu Xue*, Meihua Chen*

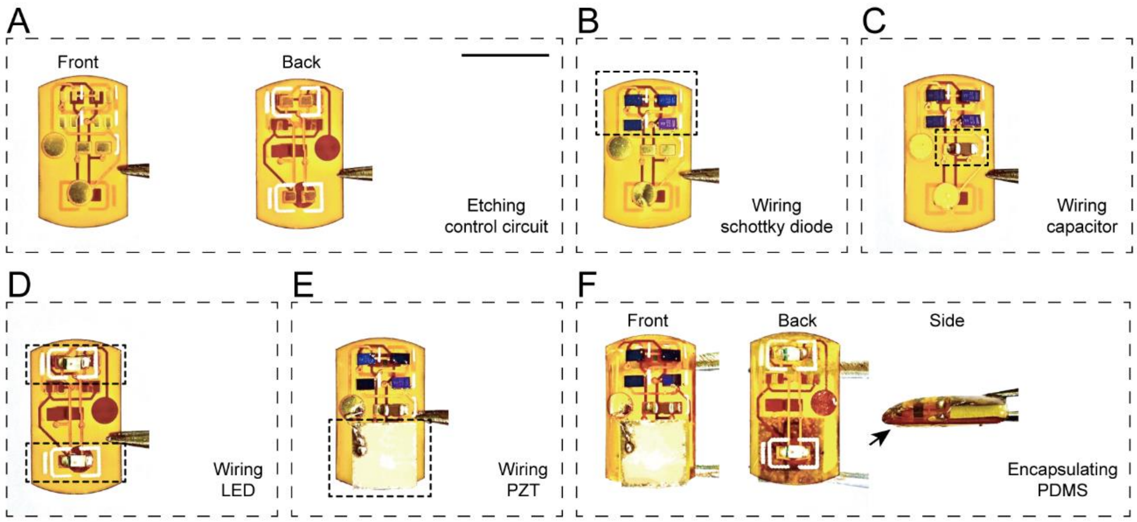

Figure S1. The manufacturing process of therapeutic tablet. Scale bar, 5 mm.

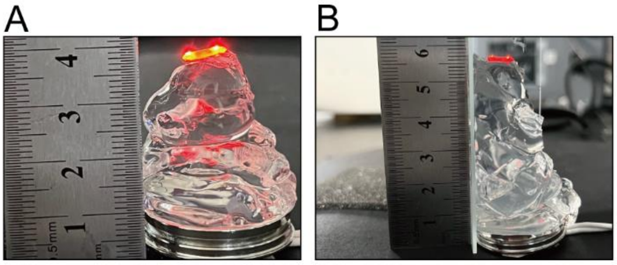

Figure S2. The working state of the therapeutic tablet and ultrasonic generator at different distances. (A) The working state of the therapeutic tablet and ultrasonic generator at 4 centimeters (cm). (B) The therapeutic tablet can still work stably at 6 cm distance with ultrasonic generator.

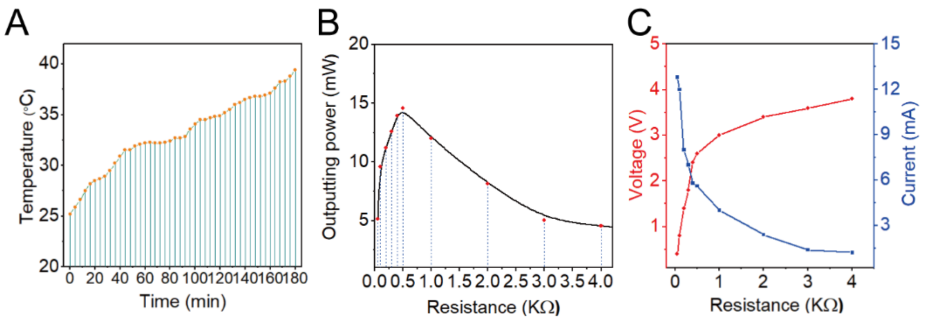

Figure S3. The characterization of therapeutic tablet during operation. (A) Changes in temperature of therapeutic tablets at different working hours. The temperature of the device was at 28.5℃, 30.9℃, 32.2℃, and 34.9℃ at a treatment duration of 20 min, 40 min, 60 min, and 120 min without significant thermal damage or side effects. (B) The output power of the unit was related to the load resistance of the circuit. (C) The corresponding output voltage and current with the load resistance of the circuit.

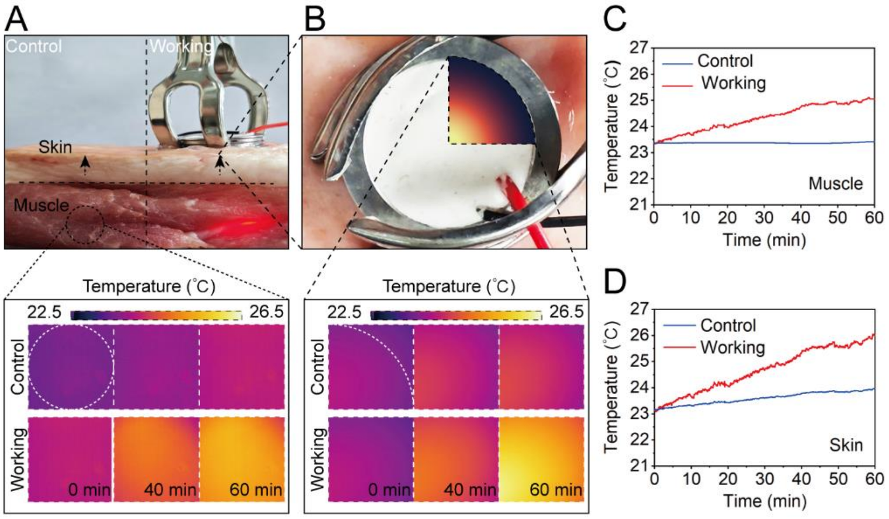

Figure S4. Temperature changes in the working process of ultrasound-driven therapeutic tablets in pork tissue. (A) Working status of the therapeutic tablet and thermal imaging of temperature changes. (B) Temperature measurement diagram of the ultrasound probe and thermal imaging of local skin tissue. (C) The temperature change curve of local muscle tissue after one hour of therapeutic tablet operation. (D) The temperature change curve of local skin tissue after one hour of ultrasound probe operation.

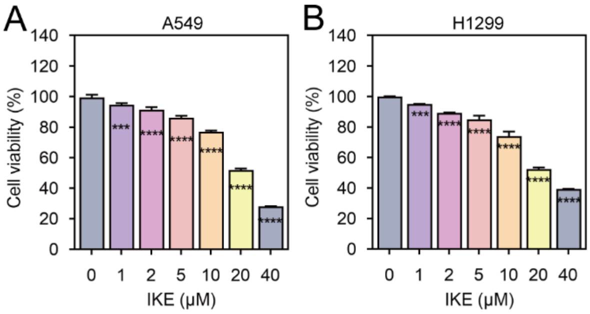

Figure S5. Effect of different concentrations of the ferroptosis inducer IKE on the activity of human lung cancer cells. The non-small cell lung cancer cell (NSCLC) lines A549 and H1299 were treated with different concentrations of IKE for 24 hours and cell viability was detected using CCK-8 assay. At 20 μM, the cell viability of A549 (A) and H1299 (B) were decreased to 52.0% and 52.4%, respectively. Data are presented as means ± SEM, $n = 4$. Ordinary one-way ANOVA with multiple comparisons, $^{***}P \leq 0.001$; $^{****}P \leq 0.0001$.

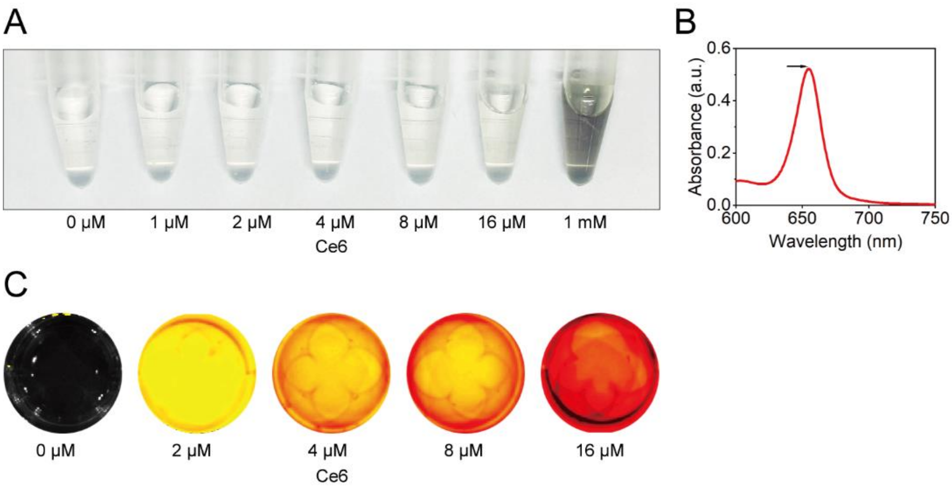

Figure S6. Dissolution and absorbance spectrum of Ce6. Ce6 was dissolved in cell-grade DMSO to a concentration of 10 mM and then diluted with PBS or cell culture medium to obtain Ce6 working solutions of different concentrations. (A) Photographs of Ce6 at different concentrations diluted in PBS. (B) Absorbance spectrum of Ce6. (C) Fluorescence images of Ce6 at different concentrations diluted in cell culture medium.

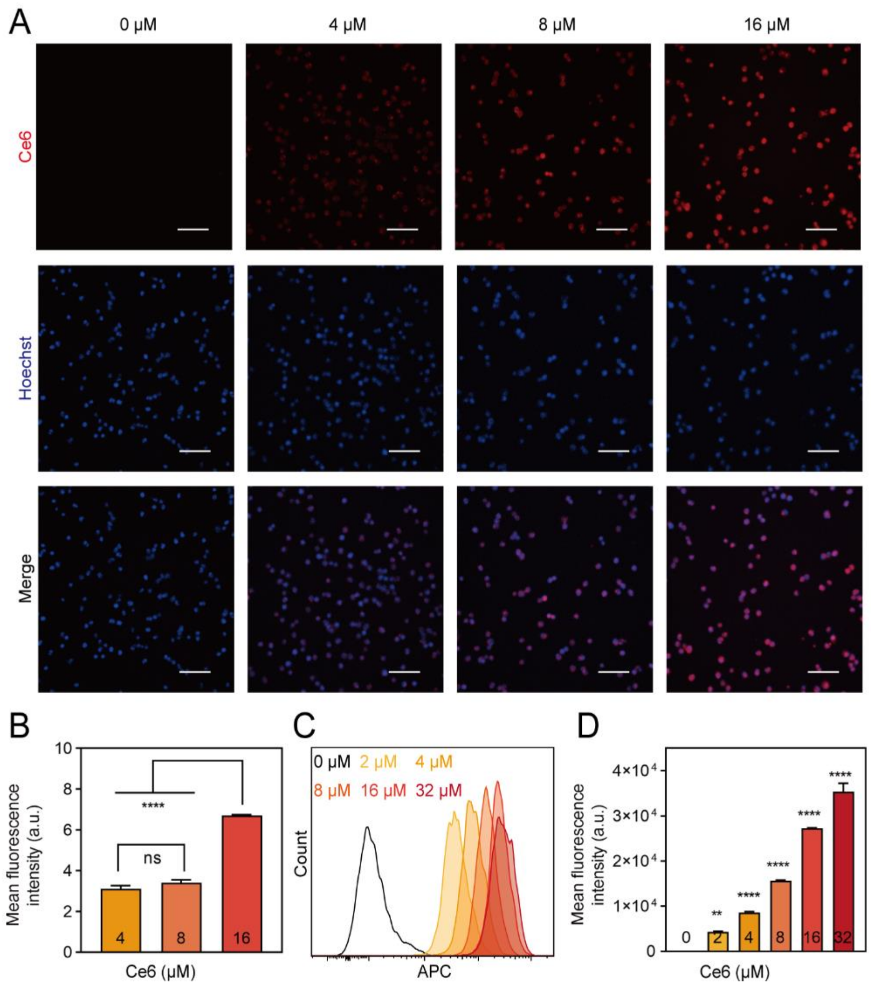

Figure S7. Absorption of Different Concentrations of Ce6. 1×10^5 LLC cells were incubated in the dark with 2, 4, 8, 16, and 32 μM Ce6 prepared in serum-free DMEM for 2 hours, followed by confocal imaging and flow cytometry. (A) and (B) show the fluorescence images and corresponding statistical analysis of Ce6 treatment at different concentrations. Scale bars, 50 μm. (C) and (D) show the flow cytometry results and corresponding statistical analysis of Ce6 treatment at different concentrations. Data are presented as means ± SEM, n = 3. Ordinary one-way ANOVA with multiple comparisons, not significant (ns), $P \geq 0.05$; $^{**}P \leq 0.01$; $^{****}P \leq 0.0001$.

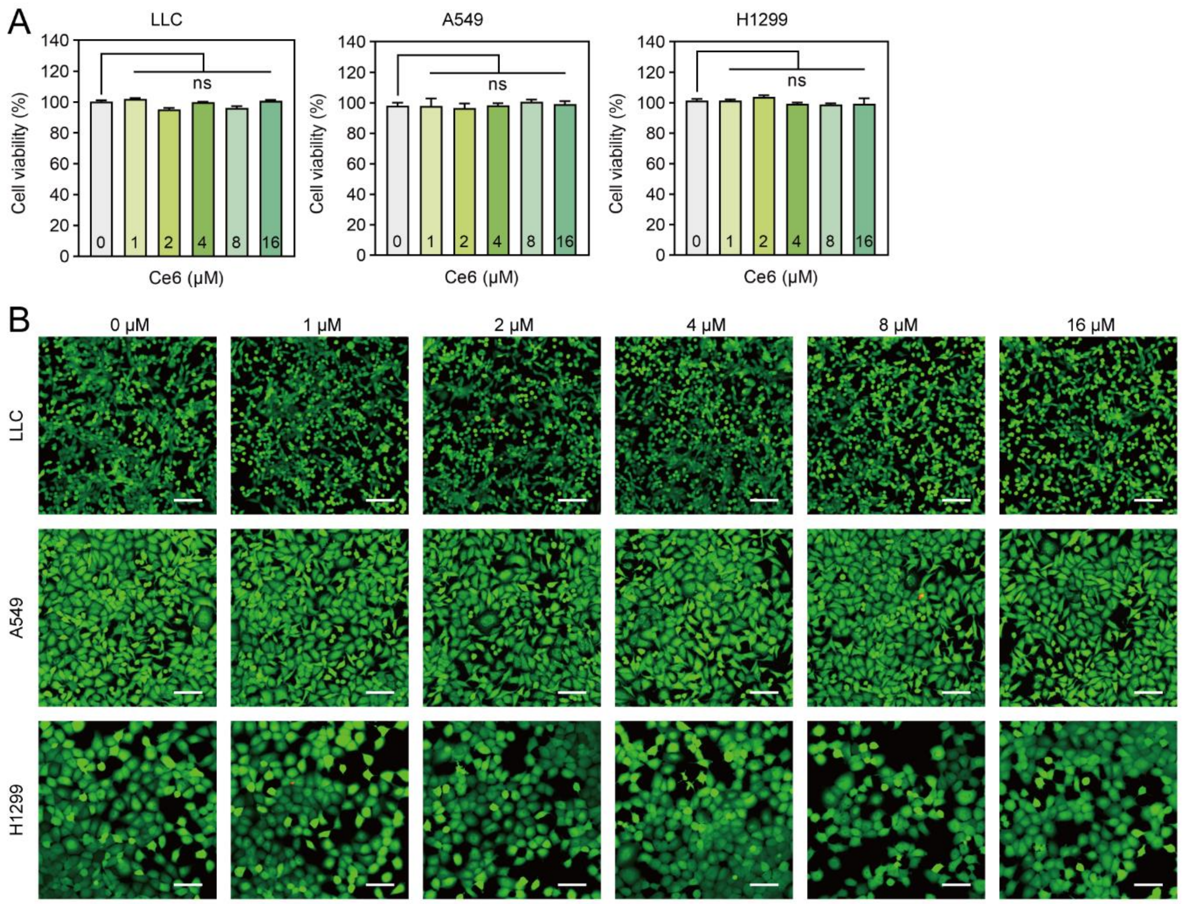

Figure S8. Evaluation of the biotoxicity of Ce6. Lung cancer cell lines were seeded and incubated for 24 hours. After that, the cells were treated with different concentrations of Ce6 and incubated in the dark for 6 hours. Following a further 24-hour incubation, the cell viability was assessed using the CCK-8 assay (A) and the live and dead cells were stained using Calcein-AM/PI fluorescence staining (B), scale bar = 100 μm. Data are presented as means ± SEM, *n* = 4. Ordinary one-way ANOVA with multiple comparisons, not significant (ns).

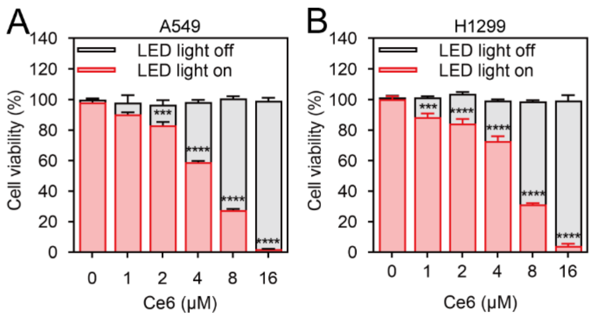

Figure S9. Effect of different concentrations of photosensitizers Ce6 on the viability of lung cancer cells. NSCLC cells were treated with different concentrations of the photosensitizer Ce6, with or without 20 min of light, and cell activity was detected by CCK-8 after 24 h. In the absence of light, Ce6 was not activated and thus affected A549 (A) and H1299 (B). While under the activation of 660 nm red light, the higher the Ce6 concentration, the greater the killing for the cells. When the Ce6 concentration was 8μM, the cellular viability of A549 and H1299 decreased by nearly 70%. Data are presented as means ± SEM, n = 4. Ordinary one-way ANOVA with multiple comparisons, $^{***}P \leq 0.001$; $^{****}P \leq 0.0001$.

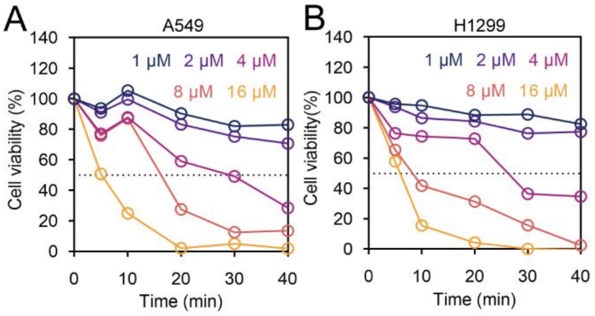

Figure S10. Effect of different concentration of photosensitizer and treatment durations of PDT on cellular viability. PDT was performed for 10 min, 20 min, 30 min, and 40 min with 1 μM, 2 μM, 4 μM, 8 μM, and 16 μM Ce6, and cell viability was detected by CCK-8 after 24 h. The antitumor effect of PDT is closely related to the dosage of the photosensitizer and duration. The higher the concentration and the longer the treatment time, the more obvious the antitumor effect. When the photosensitizer concentration was 8 μM, the cell activity of A549 (A) and H1299 (B) decreased to 27.59% and 31.3% when PDT was applied for 20 min. Data are presented as means ± SEM, *n* = 4.

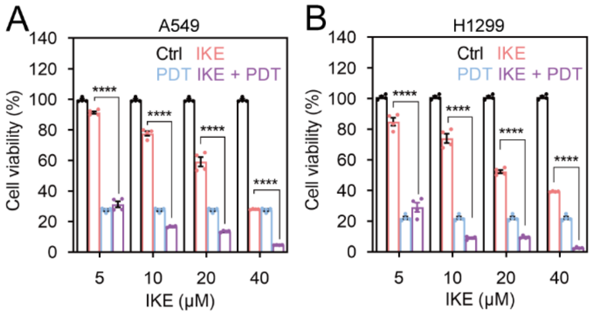

Figure S11. In vitro antitumor activity of IKE in combination with PDT therapeutic tablet. NSCLC cells were treated with PDT (Ce6: 8 μM, times: 20 min) mixed with various concentrations of IKE, and CCK-8 was utilized to measure cell viability 24 hours later. In the 10 μM IKE, IKE plus PDT treatment group, the cellular viability of A549 (A) and H1299 (B) decreased by about 85% and 90%, respectively. Data are presented as means ± SEM, $n = 4$. Ordinary one-way ANOVA with multiple comparisons, $^{****}P \leq 0.0001$.

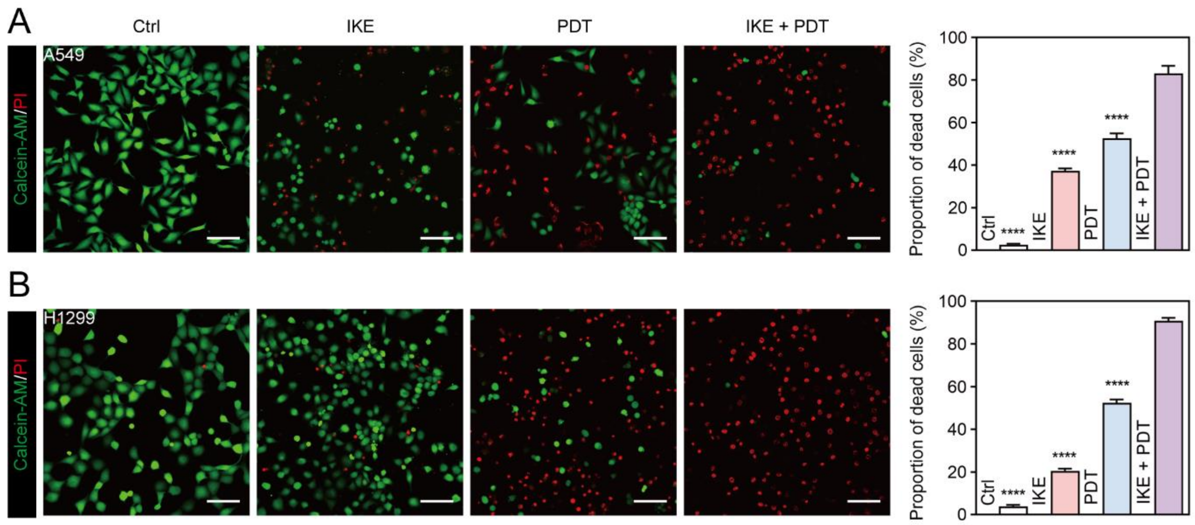

Figure S12. In vitro antitumor activity of IKE in combination with PDT therapeutic tablet. After 24 hours of varied treatments, NSCLC cells were stained with calcein acetoxymethyl ester (Calcein-AM, staining living cells with green fluorescence) and Propidium Iodide (PI, staining dead cells with red fluorescence). Both A549 (A) and H1299 (B) detect higher red fluorescence in the IKE + PDT group, indicating that there are more dead cells. Statistics indicate that up to 80% of cells in the combination therapy group had died. Scale bars, 50μm. Data are presented as means ± SEM, n = 4. Ordinary one-way ANOVA with multiple comparisons, $^{****}P \leq 0.0001$.

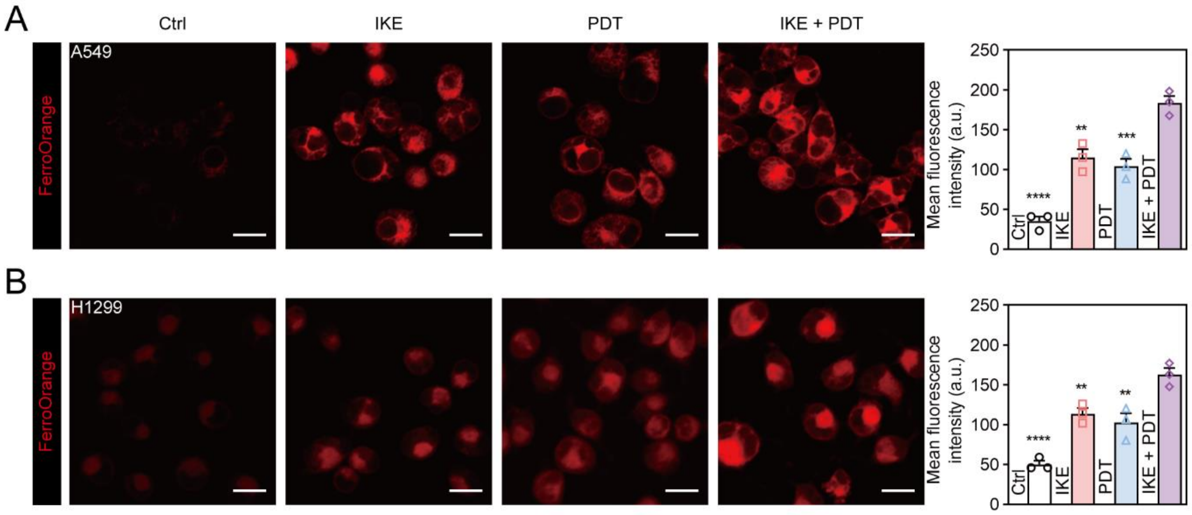

Figure S13. IKE combined with PDT increases the level of intracellular ferrous ions in lung cancer cells. Intracellular ferrous ions of A549 (A) and H1299 (B) was measured via FerroOrange fluorescence staining. The level of intracellular ferrous ions in the hybrid therapy group was increased. Scale bars, 20 µm. Data are presented as means ± SEM, n = 3. Ordinary one-way ANOVA with multiple comparisons, $^{**}P \leq 0.01$; $^{***}P \leq 0.001$; $^{****}P \leq 0.0001$.

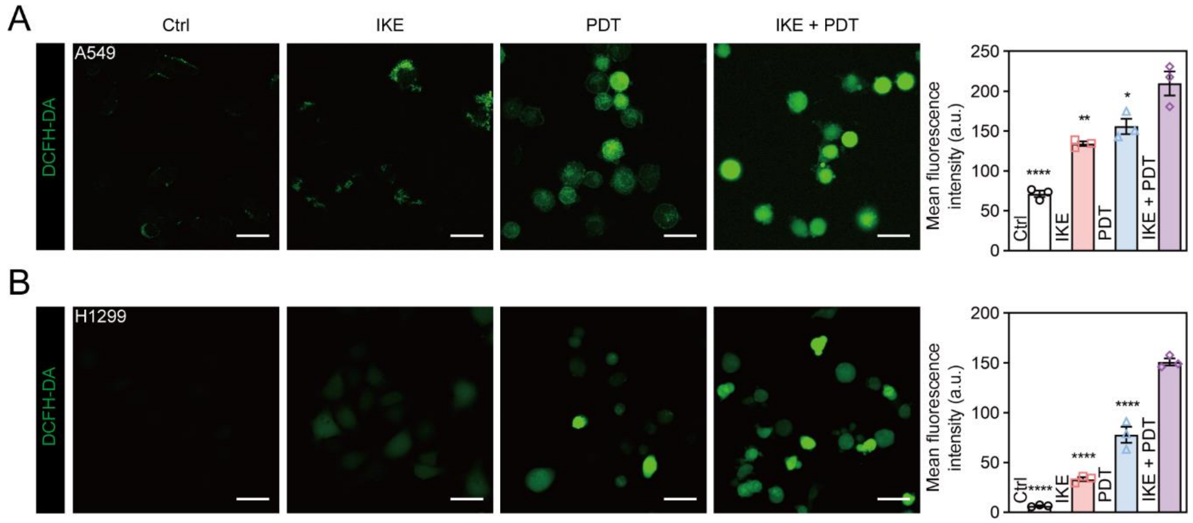

Figure S14. IKE combined with PDT increases the level of ROS in lung cancer cells. After A549 (A) and H1299 (B) were treated with ctrl, IKE, PDT, and IKE + PDT for 24 hours, intracellular ROS were detected and statistically analyzed using 2',7'-dichlorodihydrofluorescin diacetate (DCFH-DA) probe. The stronger the green fluorescence, the more intracellular ROS was produced. The level of ROS in the hybrid therapy group was significantly increased. Scale bars, 50 µm. Data are presented as means ± SEM, n = 3. Ordinary one-way ANOVA with multiple comparisons, $^{*}P < 0.05$; $^{**}P \leq 0.01$; $^{***}P \leq 0.001$; $^{****}P \leq 0.0001$.

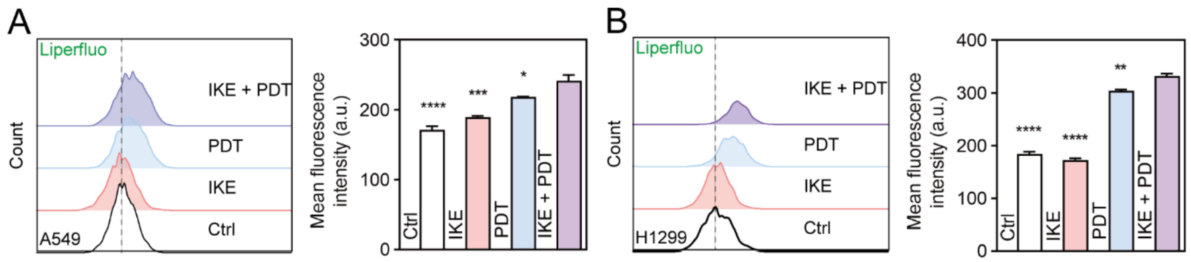

Figure S15. IKE combined with PDT increases the level of lipid peroxide in lung cancer cells. After A549 (A) and H1299 (B) were treated with ctrl, IKE, PDT, and IKE + PDT for 24 hours, intracellular lipid peroxide was detected and statistically analyzed using liperfluo fluorescence probe by flow cytometry. The stronger the mean fluorescence intensity of FITC, the more intracellular lipid peroxide was produced. The level of lipid peroxide in the hybrid therapy group was increased. Scale bars, 50 µm. Data are presented as means ± SEM, $n = 3$. Ordinary one-way ANOVA with multiple comparisons, $^{*}P < 0.05$; $^{**}P \leq 0.01$; $^{***}P \leq 0.001$; $^{****}P \leq 0.0001$.

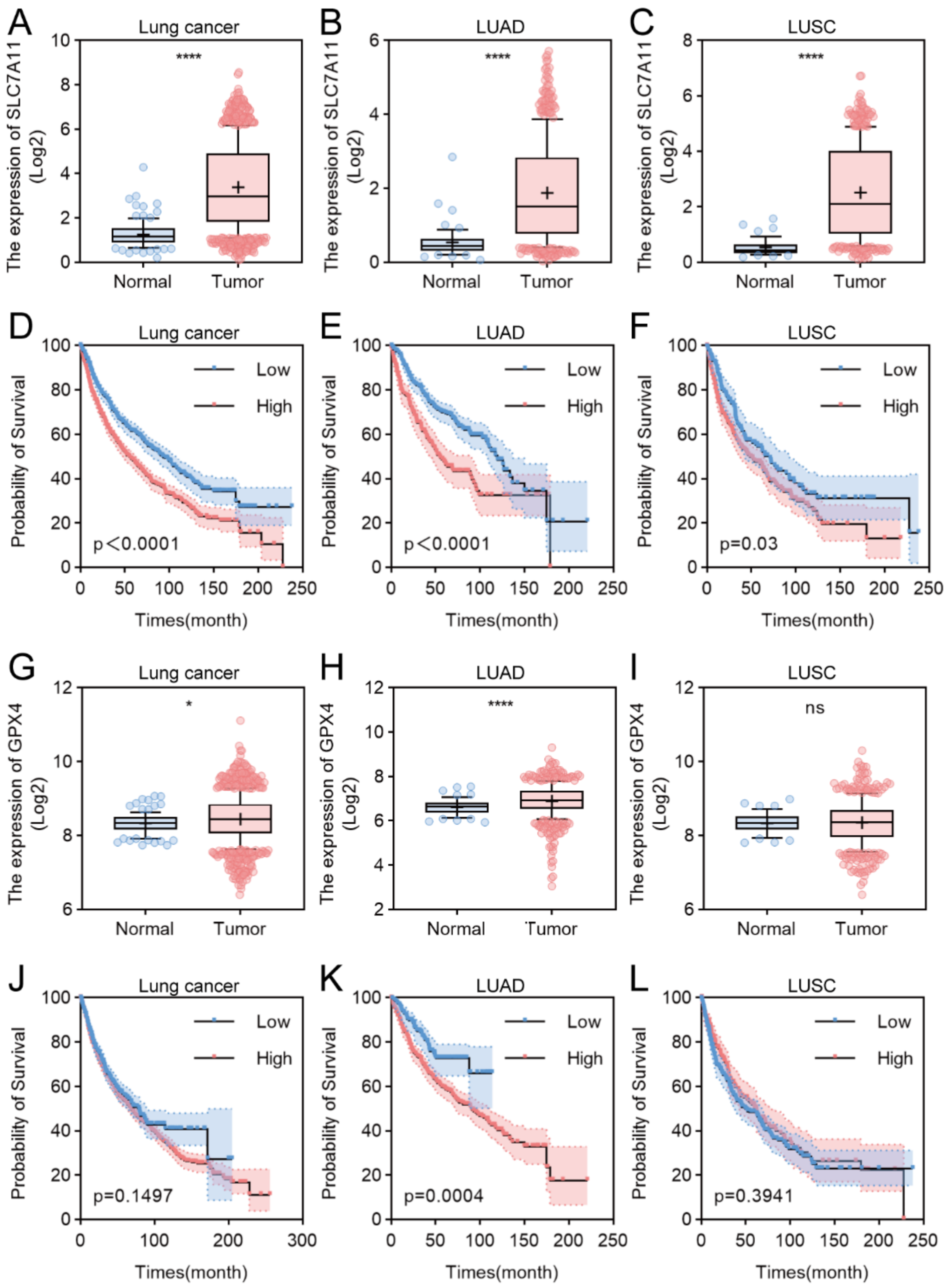

Figure S16. SLC7A11 and GPX4 are potential tumor promoter in lung cancer. (A), (B), and (C) SLC7A11 mRNA expression was investigated in The Cancer Genome Atlas (TCGA) database and compared between lung cancer samples and normal mammary tissues. (D), (E), and (F) The association of SLC7A11 with overall survival (OS) was analyzed in Kaplan Meier plotter website. (G), (H), and (I) GPX4 mRNA expression was investigated in TCGA database and compared between lung cancer samples and normal mammary tissues. (J), (K), and (L) The association of GPX4 with OS was analyzed in Kaplan Meier plotter website.

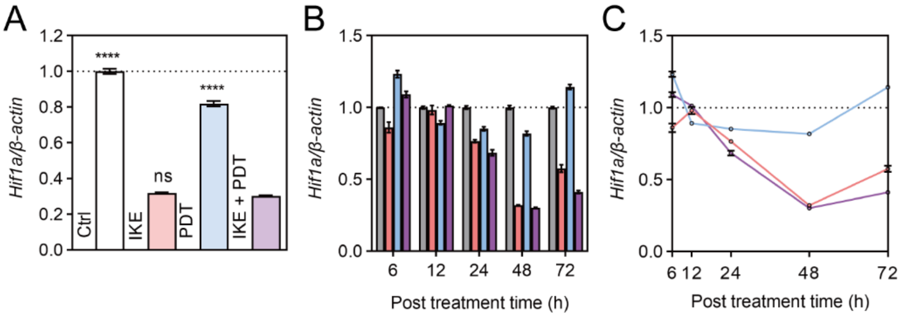

Figure S17. Hypoxia-inducible factor 1-alpha (*Hif1a*) dynamics after different treatments. 6 h, 12 h, 24 h, 48 h, and 72 h after treatment, real-time fluorescence PCR was utilized to detect LLC in various treatment groups. *β-actin* was used as housekeeping gene and relative expression of the target gene was determined using the $2^{-\Delta\Delta Ct}$ method. In the IKE and IKE + PDT groups, *Hif1a* gene, which reflect intracellular oxygen levels, were significantly reduced, and intracellular hypoxia was improved.

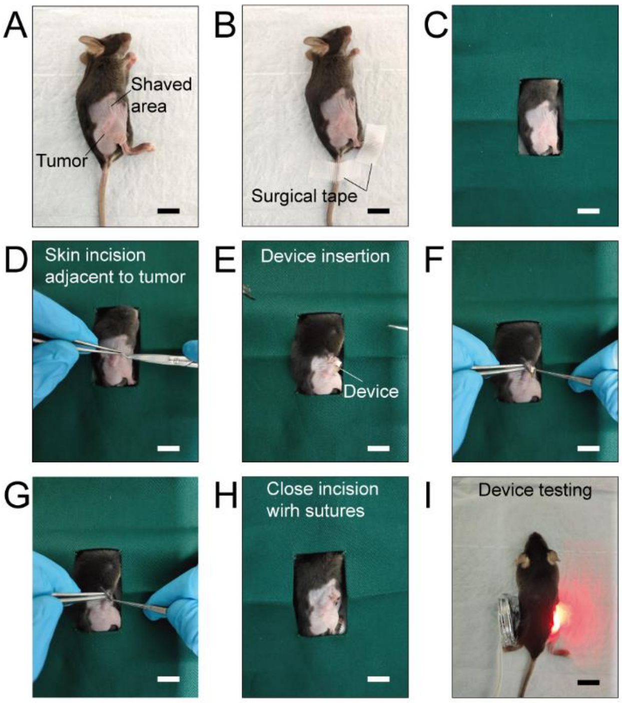

Figure S18. Surgical procedure for the implantation of the therapeutic tablet. (A) The tumor area is shaved. (B) the hind legs and tail fixated with surgical tape. (C) The body is covered by a surgical drape. (D) Skin incision is made adjacent to the tumor. (E) to (G) The device is fixed in place by suturing flaps to the skin. (H) The incision is closed by suturing. (I) the device tested by wireless powering over the skin. Scale bar, 1 cm.

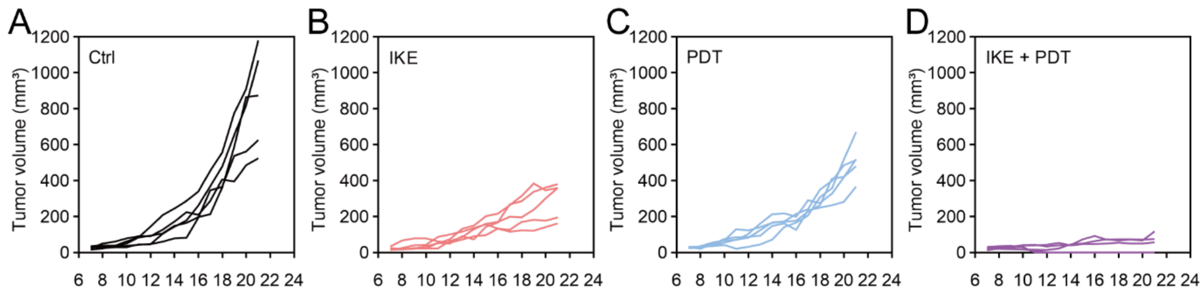

Figure S19. Tumor growth curves of individual mice with different treatments.

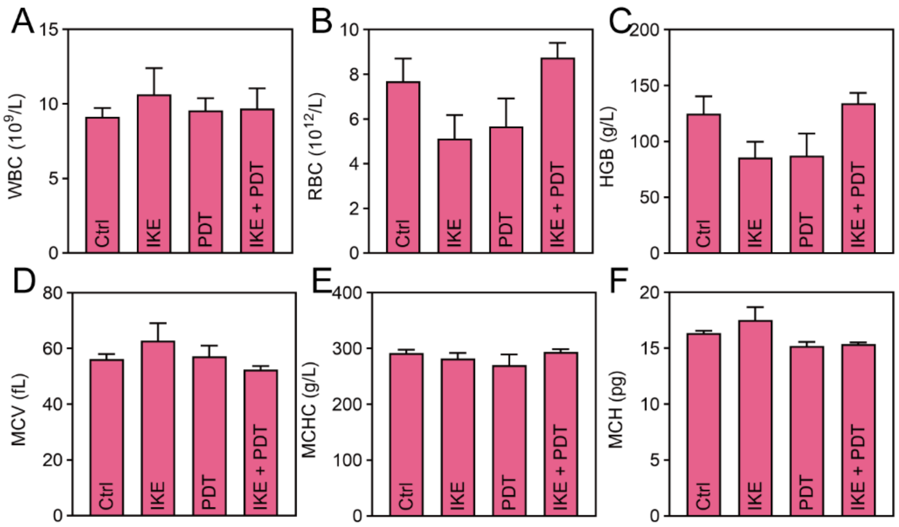

Figure S20. Blood routine of mice after treatment.

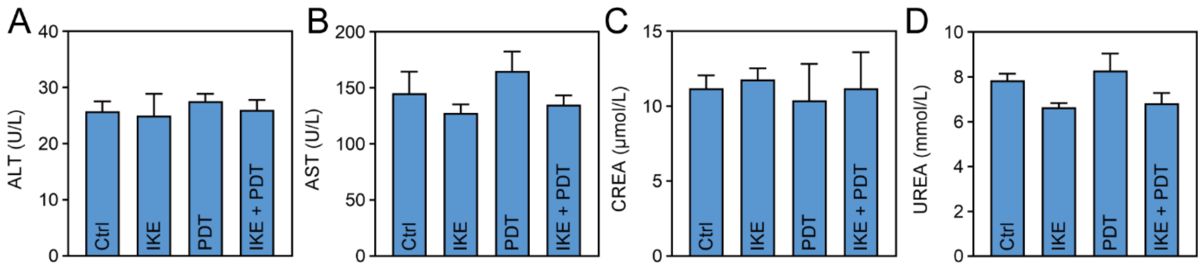

Figure S21. Biochemistry of mice after treatment.

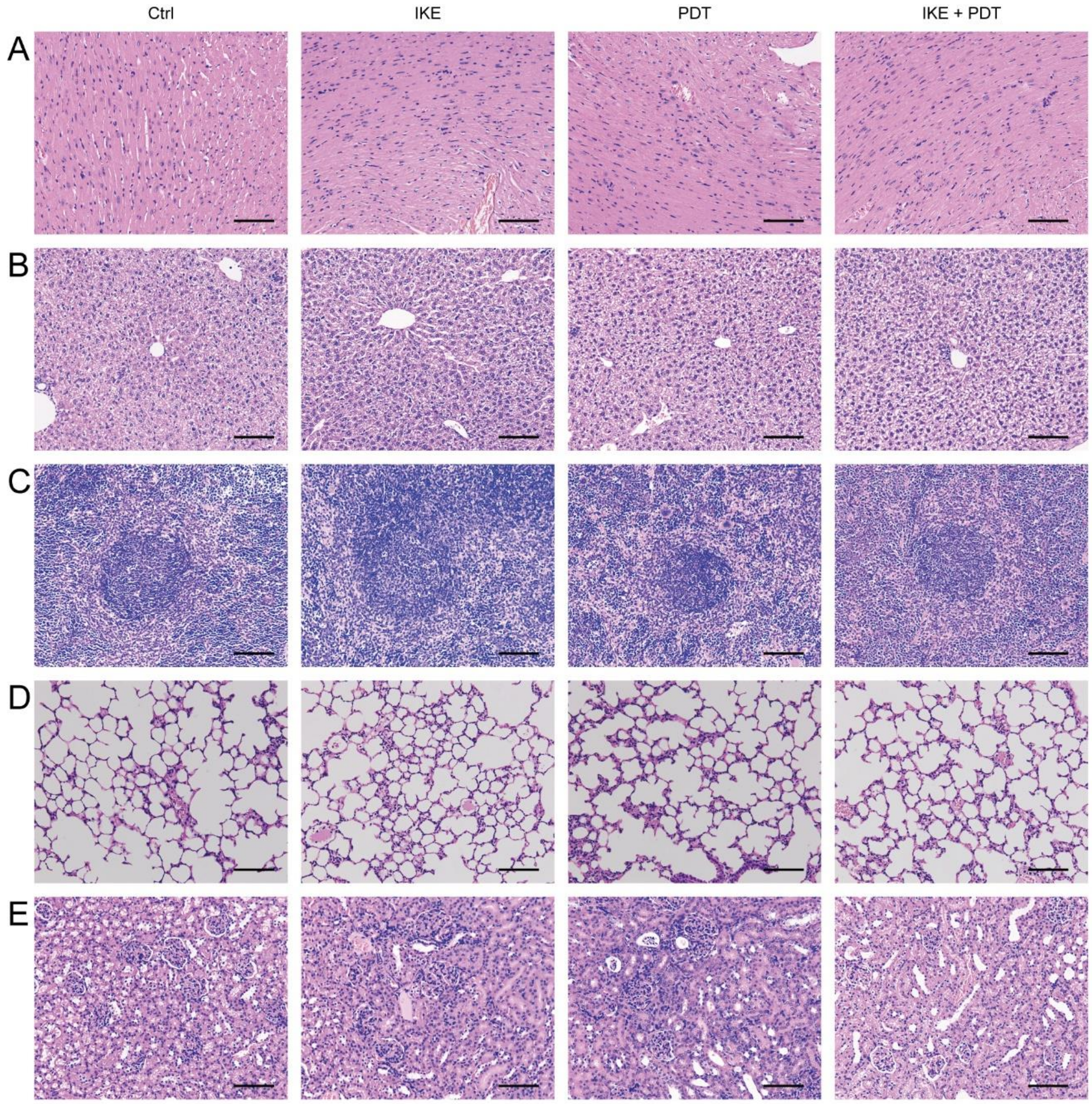

Figure S22. Histological morphology of mice major organs after treatment. H&E staining of mice major normal organ including heart (A), liver (B), spleen (C), lung (D), and kidney (E) after various treatments. Scale bars, 100 μm.

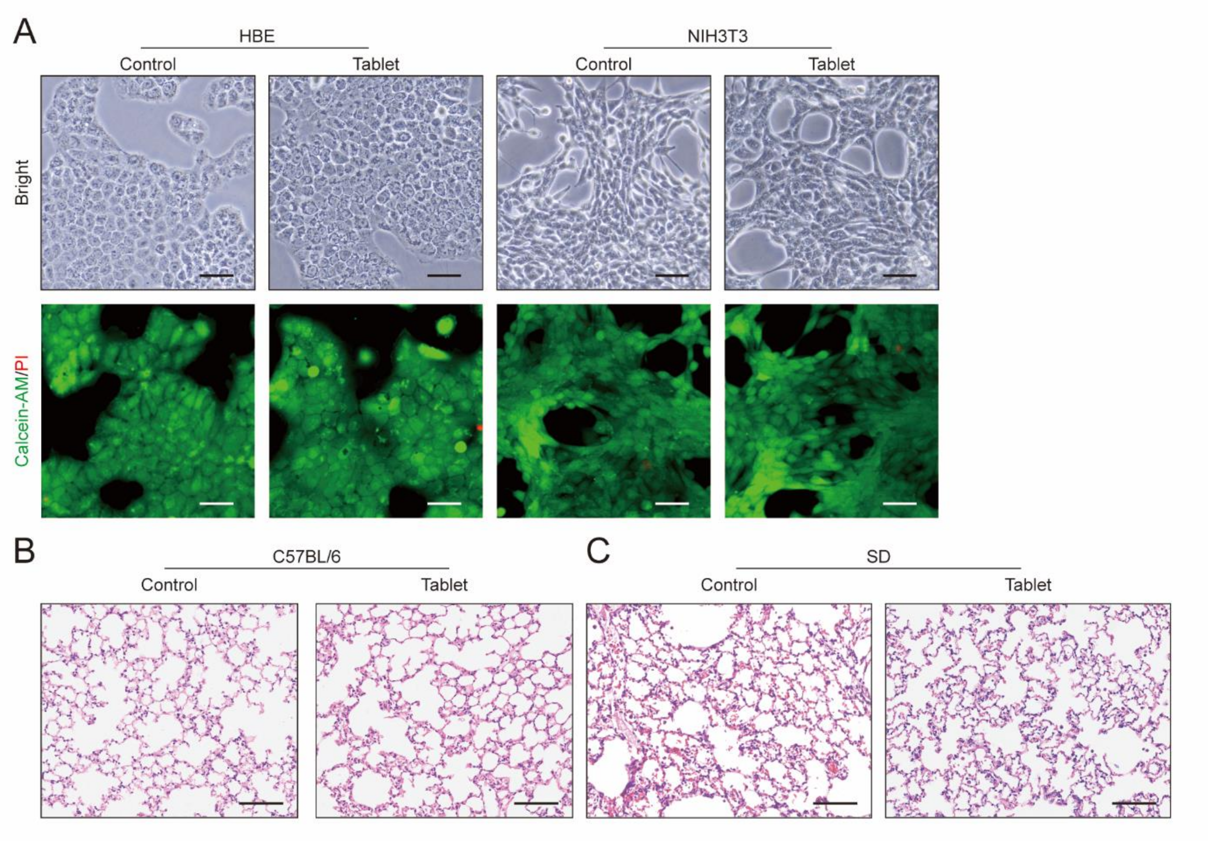

Figure S23. The biological safety assessment of therapeutic tablet. (A) After 24 hours of co-cultivating therapeutic tablet with HBE and NIH3T3, Calcein-AM/PI staining was performed. Scale bar, 50μm. (B) Three days after implanting therapeutic tablet into the thoracic cavity of C57BL/6 mice, HE staining was performed on lung tissue. Scale bar, 100μm. (C) Three days after implanting therapeutic tablet into the thoracic cavity of SD rat, HE staining was performed on lung tissue. Scale bar, 100μm.

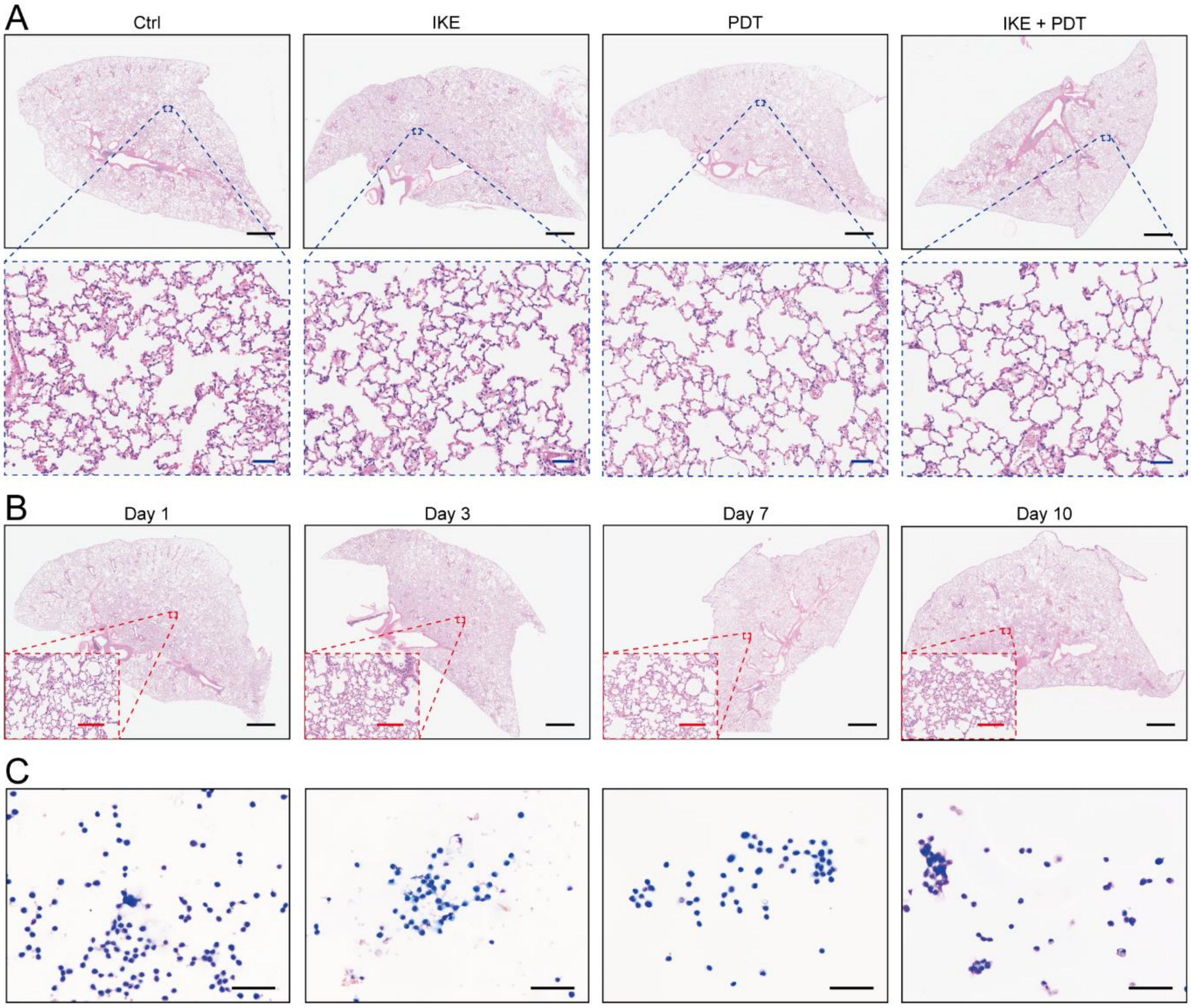

Figure S24. Biological safety evaluation of therapeutic tablet on rat lung tissue. (A) Lung tissue HE staining under different treatment conditions. Scale bar, 2.5 mm (black), 50 μm (blue). (B) Changes in lung tissue on the 1st, 3rd, 7th, and 10th day after treatment with the IKE + PDT group. Scale bar, 2.5 mm (black), 150 μm (red). (C) Microscopic examination results of bronchoalveolar lavage fluid images at the above time points. Scale bar, 50 μm.

Movie S1.

A recording of the therapeutic tablet at work in a subcutaneous tumor mouse model.

Movie S2.

A recording of the therapeutic tablet at work in the thoracic cavity of rat.

Movie S3.

A recording of the therapeutic tablet at work in a in situ tumor mouse model.
